# Variable renewables fortify Ecuador’s power system against recurrences of drought-driven energy crises

**DOI:** 10.1038/s44221-026-00617-w

**Published:** 2026-04-07

**Authors:** Sebastian Sterl, Luis E. Pineda, Tinne Mast, Yuliana Rodríguez, Paul Muñoz, Wim Thiery

**Affiliations:** 1 Department of Water and Climate, Faculty of Engineering, Vrije Universiteit Brussel, Brussels, Belgium; 2 Directorate-General for Energy, European Commission, Brussels, Belgium; 3 School of Earth Sciences, Energy and Environment, Universidad Yachay Tech, Urcuquí, Ecuador; 4 VITO/EnergyVille, Genk, Belgium; 5 Department of Chemical Engineering, University of Patras, Patras, Greece; 6 Departamento de Recursos Hídricos, Facultad de Ingeniería Agrícola, Universidad de Concepción, Chillán, Chile

**Keywords:** Energy modelling, Hydroelectricity, Renewable energy, Hydrology, Energy infrastructure

## Abstract

In 2023–2024, two successive failed rainy seasons plunged hydropower-dependent Ecuador into an unprecedented energy crisis. As rivers ran dry and hydropower generation dwindled, the Ecuadorian government had to implement daily nationwide electricity cuts throughout 2024 to keep reservoirs from emptying completely. In this Article, we show that opting for a strong build-out of solar and wind power—often criticized for their variability and purported lack of dependability—could become an important element in the fortification of Ecuador’s power system against similar future droughts. Exploiting a newly identified resource complementarity, dubbed extreme-year synergy, a coupling of hydropower operation with solar and wind energy generation would safeguard reservoir levels in critical periods, largely compensate the dry-year hydropower deficit, and substantially reduce the need for thermal backup capacity and generation, quantified through a new concept, the ‘indirect’ capacity credit of variable renewables. The identified opportunities have ramifications for hydro-dependent nations across Latin America and elsewhere.

## Abstract (web summary)

Ecuador’s reliance on hydropower led to an energy crisis following two failed rainy seasons, highlighting the need for alternative solutions. Here the authors demonstrate that integrating solar and wind power with hydropower, leveraging ’extreme-year synergy’, can stabilize energy supply during droughts, offering a model for increased power system resilience in hydro-dependent regions globally.

## Main

**Fig. 1:**
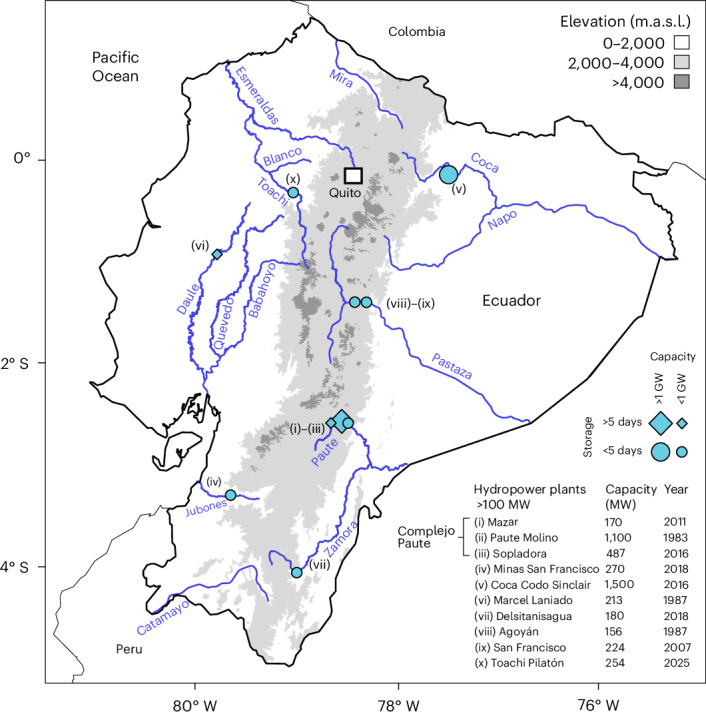
Ecuador’s main hydropower plants. Map showing the location of Ecuador’s hydropower plants of >100-MW rated capacity, distributed on both sides of the Andean mountain range (indicated by contour lines at 2 and 4 km of altitude). The hydropower plants are subdivided according to their capacity (large/small icons: installed capacity above/below 1 GW) and reservoir storage (diamonds/circles: more/less than 5 days of average river discharge). The main focus here is the Paute hydroelectric complex, or Complejo Paute, that is hydropower plants (i)–(iii). The column ‘Year’ refers to entry into service. m.a.s.l., metres above sea level. Basemap from the Database of Global Administrative Areas (GADM) v.4.1 (https://gadm.org/data.html) with river shape data from Natural Earth (https://www.naturalearthdata.com/downloads/10m-physical-vectors/10m-rivers-lake-centerlines) and the Humanitarian OpenStreetMap Team (https://data.humdata.org/dataset/hotosm_ecu_waterways) and elevation data from the Instituto Geográfico Militar (https://www.geoportaligm.gob.ec/portal/index.php/cartografia-de-libre-acceso-escala-50k) and the NASA Shuttle Radar Topography Mission (https://www.earthdata.nasa.gov/data/catalog/lpcloud-srtmgl1-003). The Galápagos archipelago is not shown.

For over a decade, Ecuador has opted to rely on hydropower to meet its increasing electricity demand. Its power system has expanded from around 6 GW in 2015 to 9 GW in 2025, primarily by adding hydropower plants, and the average share of hydropower in the electricity mix now lies at around 70% (ref. ^1^), the remaining 30% being almost exclusively sourced from oil- and gas-fired power plants. Bisected from north to south by the Andes mountains (Fig. 1), from which rivers drop steeply eastwards to the Amazon basin and westwards to the Pacific Ocean, Ecuador’s topography indeed seems well-suited to a hydro-based power system. Its current hydropower fleet is divided across both sides of the Andean range, allowing the spatiotemporal synergies between the interlinked but climatically distinct Amazonian and Pacific rainfall regimes to be exploited, with river discharges peaking several months later on the Amazonian side compared with on the Pacific side^2–4^.

**Fig. 2:**
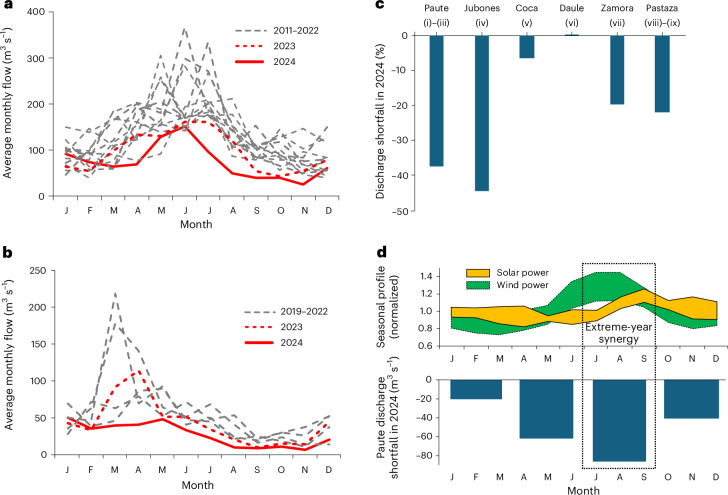
The 2023–2024 drought and its effects on resource complementarity. **a**, Average monthly discharge of the Paute River (Amazonian drainage basin), housing the Complejo Paute (plants (i)–(iii) in Fig. 1). **b**, Average monthly discharge of the Jubones River (Pacific drainage basin), housing Minas San Francisco (plant (iv) in Fig. 1). **c**, The discharge shortfall in 2024 relative to the historical average for all rivers housing active hydropower plants with a capacity of >100 MW (the numerals refer to the hydropower stations shown in Fig. 1, located on the given rivers). **d**, Seasonal profiles of solar and wind capacity factors (top, 2011–2024 envelopes, normalized by the average across the same period) compared with quarterly Paute River flow shortfall in 2024 relative to the average conditions for 2011–2023 (bottom). The discharge data are from the power utility Corporación Eléctrica del Ecuador (CELEC) for the Paute and Jubones rivers^52^ or otherwise from the grid operator Centro Nacional de Control de la Energía (CENACE)^51^; VRE profiles are from refs. ^79,80^ (see Methods).

Large parts of South America were hit by two successive dry years during the 2023–2024 El Niño event, including Ecuador^5^. Relatively low discharge of the Paute River in 2023 drained hydropower reservoir storage in the Paute hydroelectric complex (Complejo Paute; Fig. 1), a single-purpose hydropower cascade that hosts the majority of Ecuador’s flexible storage reserves. Towards the end of 2023, reservoirs in the Complejo Paute were already close to critical levels. The situation worsened when 2024 turned out to be even drier, with the seasonal Amazonian–Pacific complementarity disappearing in southern Ecuador (Fig. 2a,b) and many rivers experiencing major discharge shortfalls (Fig. 2c). An electricity crisis ensued that lasted the entire year, forcing the government to implement daily blackouts of up to 12 hours to prevent reservoirs from becoming completely inoperational. The situation was described as ‘going back to the eighteenth century’ as the blackouts caused vast economic damage and human suffering^6^. The crisis provisionally ended in early 2025 when normal rainfall returned. By then, it was clear that Ecuador’s hydro dependency was a major liability in the face of droughts.

## Hybridizing hydropower with variable renewables

For hydro-dependent countries, the hybridization of existing hydropower with new solar and wind power (hereafter called variable renewables (VREs)) has repeatedly been suggested as a route towards power mix diversification. Such hybridization involves gradually adapting the operation of the reservoir-based hydropower fleet to provide flexibility to support the grid integration of VREs, keeping more water stored in reservoirs during periods of high VRE penetration and releasing more water in periods of low sunshine and wind. In the process, elegant spatial, diurnal and seasonal synergies between hydropower and VREs can be harnessed^7^. In South America, Uruguay is already considered a success story in hydro-supported VRE expansion^8,9^ and further opportunities have been highlighted, for example, for Suriname^10^, Brazil^11,12^, the MERCOSUR subregion consisting of Argentina, Brazil, Chile, Paraguay and Uruguay^13^ and at the continent level^14,15^. Studies have also identified considerable potential in Africa, for example, for Ethiopia^16^, West Africa^7^, and East and Southern Africa^17^, as well as in (South)-East Asia^18^, particularly China^19–22^ and other Mekong basin countries^23,24^.

Given the storage capacity on the Paute River, hydro-supported VRE expansion could be an option for Ecuador too. However, this remains understudied; some (pre-crisis) studies on Ecuador, while acknowledging the potential benefits of diversification, suggested continued hydro-dominance as being cost-optimal^25,26^, while, more recently, floating VRE plants on Ecuadorian hydro reservoirs have been proposed as a diversification strategy^27,28^, but without investigating the implications for hydropower operation. Ecuador’s predominant focus on hydropower has been identified as a barrier in itself to VRE adoption, with hydropower having been the dominant recipient of public funding compared with other power generation technologies in the recent past^29^.

Ostensibly, the seasonal hydro–VRE synergies identified in many of the aforementioned cases seem to be absent in Ecuador. Seasonal VRE generation potential (Fig. 2d, top) is not particularly anticorrelated with Paute River discharge (Fig. 2a): the seasonal maxima of wind (July–August) and solar (September) production potential occur shortly after the discharge peak (June–July), and the low discharge period (October–December) does not coincide with noteworthily strong VRE resources. However, this absence of correlation in regular years spells opportunities for extreme years: VREs reach peak potential in precisely the period in which Paute River discharge showed the strongest shortfall in 2024 (Fig. 2d, bottom). As the same trend appears across historical drought periods, and as, from a climatological perspective, VRE potential in the region is demonstrably uncorrelated to droughts^15^ (Supplementary Note 1), this ‘extreme-year synergy’ seems robust and may hold opportunities for power system resilience in Ecuador. Generalizing, ‘extreme-year synergy’ is defined here as a situation in which two (or more) resources exhibit similar typical seasonal profiles, with seasonal maxima tending to coincide and/or partially overlap, but in which, should one of the resources fail to exhibit its regular seasonal peak in a given year, for example, hydropower during extreme droughts, the other resources tend to maintain theirs, thus providing an opportunity to mitigate and/or partially offset the seasonal deficit resulting from the loss of the former.

Hydro–VRE synergies during extreme drought have been poorly explored, with only a few studies touching the topic in ways not necessarily relevant to Ecuador’s situation, for example, without providing details on reservoir operation^30,31^ or assuming perfect foresight^32^. Given this, in this study, we explored whether, and how, synergetic hydro–VRE operation could have helped Ecuador to weather the energy crisis, as well as the role of reservoir management therein. If Ecuador opted for large-scale VRE build-out, supported by hydropower, could it avoid reoccurrences of drought-induced energy crises?

Although we focused on Ecuador, our methodology is relevant for a broader context of challenges in power systems worldwide. Brazil was also impacted by the 2024 Amazonian drought and had to drastically reduce generation at several gigawatt-sized hydropower plants while shifting to thermal sources and imports^33,34^. In China, a record heatwave and drought in 2022 affected river flow and hydropower production in the Yangtze River basin, leading to power supply rationing and suspension of industrial activity^35–37^, as well as an increase in coal-fired power generation^38^. In Southern Africa, reduced discharge from the Zambezi River in 2023–2024 led to precariously low water levels in the reservoir of the 2-GW Kariba hydropower plant, which normally provides the bulk of Zambia and Zimbabwe’s electricity, and forced rolling blackouts of more than 20 hours a day in both countries^39^, which, in response, announced plans to increase coal-fired power capacity^40,41^. In 2024, drought conditions reduced Canada’s hydropower generation to the extent that the country, usually an exporter of electricity to the United States, became a net importer^42,43^. In 2022 and 2025, Norway faced anomalously low reservoir levels and imposed restrictions on reservoir operations and electricity exports^44,45^. Clearly, across continents, hydro-dependent countries are facing the impacts of climate variability and drought vulnerability, and investigations into the potential for hydro–VRE hybridization as a resilience measure could be of general interest.

## Modelling Ecuador’s hydropower plants

**Fig. 3:**
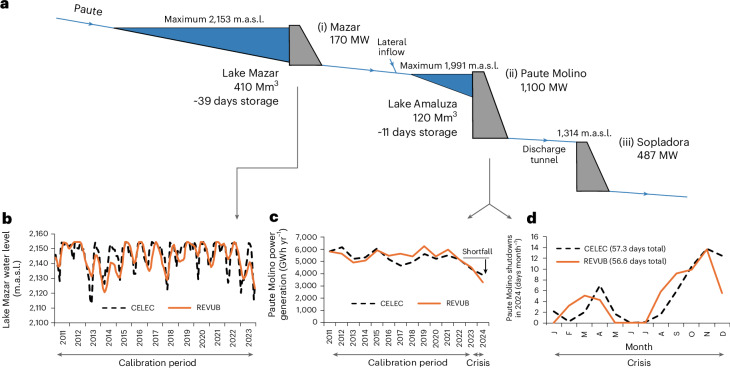
Modelling lake levels, power generation and plant shutdowns. **a**, Schematic of the Complejo Paute containing the Mazar, Paute Molino and Sopladora hydroelectric plants, as well as the Mazar and Amaluza reservoirs (inspired by ref. ^78^). Indicated are the installed capacities (MW), storage volumes (in million m^3^ (Mm^3^) and as number of days of average river discharge) and dam altitudes (m.a.s.l.). **b**–**d**, The REVUB model in this study is capable of reproducing various crucial time series of the complex during normal and extreme years, such as seasonal lake level fluctuations (in m.a.s.l.) (**b**), annual hydropower generation (in GWh yr^−1^) (**c**) and monthly accumulated duration of shutdowns in the extreme year 2024 (total number of hours in shutdown expressed in days per month) (**d**). These data are compared with historical time series data from CELEC^52^.

Our main focus was on the Complejo Paute, which houses most of the flexibility potential of Ecuador’s hydro fleet. It consists of a cascade of three hydroelectric plants (Fig. 3a): Mazar (170 MW across two turbines), Paute Molino (1,100 MW, ten turbines) and Sopladora (487 MW, three turbines). The dams of the first two plants impound reservoirs (Mazar and Amaluza) with a cumulative capacity of 530 million m^3^ (~50 days of average discharge), of which >75% is in Lake Mazar. This set-up allows the Paute Molino plant, the most powerful and flexible in the cascade, to use the regulatory capacity of both the Mazar and Amaluza lakes.

To study the hybridization of the Complejo Paute with VREs, we applied the Renewable Electricity Variability, Upscaling and Balancing (REVUB) dispatch model, an open-source software developed to study hydro-supported VRE expansion strategies and used in various studies^7,10,16,46–49^ (see Methods for details on the model and its use). In brief, the model simulates hydropower reservoir operation to meet production targets in conjunction with VREs under a range of technical and environmental constraints, from hourly to multiannual timescales. It requires a calibration period whose average river discharge is used to derive seasonal rule curves for storage drawdown and refill. We took 2011 (when Lake Mazar started operation) to 2023 as the calibration period, but simulated through to the end of 2024. Thus, the crisis year 2024 was treated as myopic: the model marches forwards in time without prior knowledge of the disastrous hydrology of 2024. This allowed a realistic simulation of what occurred: the extent of the 2024 drought had not been foreseen, so the regular operational principles of the Complejo Paute, based on historical experience, proved inadequate.

Our application of the REVUB model is capable of reproducing diverse key elements of the operation of the Complejo Paute both in regular years and during the crisis period (Fig. 3). This includes storage cycles in Lake Mazar (Fig. 3b), electricity generation by Paute Molino (Fig. 3c) and the profile of forced shutdowns of Paute Molino in 2024 (Fig. 3d). Full details of the calibration procedure, including power generation and lake-level data for all simulated plants, as well as a discussion of uncertainties, are given in Supplementary Note 2. Given the capabilities of the REVUB model, it is deemed a suitable framework to study the case of hydro-supported VRE integration in Ecuador in the context of the drought crisis.

## Effect of VREs on reservoir water storage

We designed four scenarios to investigate the effect of VRE integration on hydropower operation in the Complejo Paute, with a focus on the crisis period. The first scenario (S1) is the reference scenario (Fig. 3), without VREs. The second (S2) assumes that hydropower operation adapts to support the integration of solar power into the mix. The third (S3) assumes that hydropower supports a VRE fleet with equal capacities of solar and wind power. The fourth (S4) assumes that the solar–wind fleet is oversized with respect to the flexibility that the Complejo Paute can deliver. Across scenarios S1–S4, hydropower operation remains myopic to the 2024 drought conditions. (For details of the scenarios, see Methods, and for a geospatial representation of the VRE options, see Supplementary Note 3.)

**Fig. 4:**
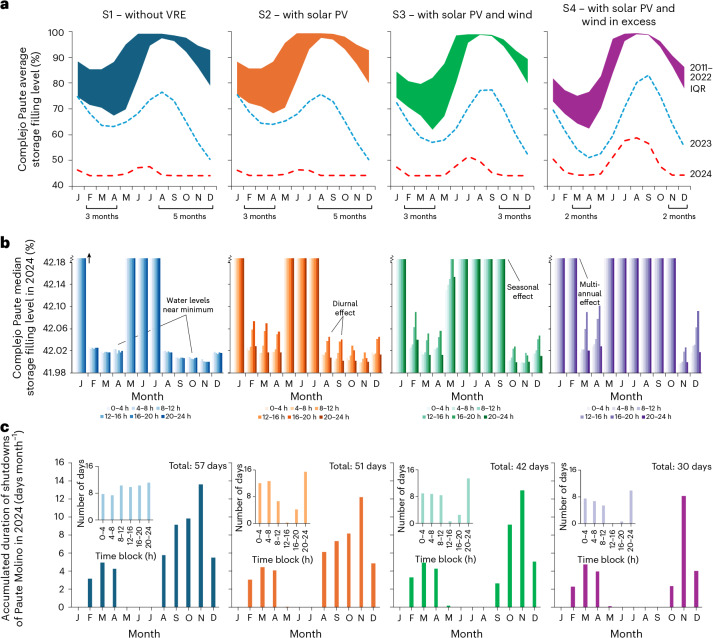
Adding VREs would help to maintain higher storage levels. **a**,**b**, Simulation outcomes showing time series of the average (**a**) and median (**b**) water storage levels in the Complejo Paute (lakes Mazar and Amaluza) under scenarios S1–S4 (from left to right). In **a**, the interquartile range (IQR) of monthly averages for the period 2011–2022 are shown, as well as the monthly averages for 2023 and 2024. The critical months in 2024, defined as having median storage levels (calculated across all hours in a month) within 0.10 percentage points of the minimum operational value (slightly below 42%), are highlighted on the horizontal axes, declining from a total of 8 months in S1 to 4 months in S4. In **b**, the median storage levels are shown for all months of 2024, divided across six different 4-hour time blocks of each day (note the upper cut-off of the *y* axis for clarity). **c**, Accumulated shutdown durations for Paute Molino in 2024 by month, declining from 57 days to 30 days under scenarios S1–S4 (left to right). Insets: accumulated shutdown durations for different time blocks.

We found that the addition of VREs would not have drastically changed normal-year drawdown/refill cycles, but could have considerably lifted water levels in late 2023 and throughout large parts of 2024 (Fig. 4a). This latter effect is not visible when hybridizing with only solar (S2, with 367 MW of solar capacity hybridizable with the Complejo Paute; Methods), but manifests strongly when adding wind (S3, with 270 MW of hybridizable capacity for solar and wind each, and S4, with 613 MW for each). The number of ‘critical months’ in 2024, when lake levels hovered around minimum operational levels and frequent plant shutdowns occurred, decreases from eight (S1 and S2) to six (S3) and four (S4). Thus, the presence of wind power allows the aforementioned ‘extreme-year synergy’ with hydropower to be harnessed (Fig. 2d), enabling reservoirs to refill relatively quickly even during an extremely weak rainy season because wind takes over high shares of the supply in the crucial refill period alongside solar.

Nevertheless, even adding only solar positively affects storage operation. The diurnal cycle of solar power generation means that hydropower can ramp down during the daytime, allowing storage levels to undergo regular ‘micro-recoveries’ (compare S2 with S1 in Fig. 4b), the individual effects of which (minor water level increases across several hours) are repeated at high frequency (that is, each day; Supplementary Note 4). Wind power adds a seasonal effect, allowing better storage of the seasonal river discharge peak (S3 in Fig. 4b), plus the secondary effect of solar–wind synergies on the day–night scale^50^, with wind typically blowing more strongly in the evenings and mornings and providing a natural complement to a hydro–solar mix (Supplementary Note 5). Last, further enlarging the solar–wind fleet reinforces those dynamics and alleviates the pressure on the hydro reservoirs further, leading to a multiannual effect in which extra water stored in 2023 as a result of hydro–VRE synergies would have allowed 2024 to start in a substantially improved position. This would have delayed the onset of the first plant shutdowns in 2024 by a few weeks and dampened their frequency (S4 in Fig. 4b). Overall, the accumulated shutdown time of the Paute Molino plant would have reduced from 57 days in S1 to 51 days in S2 (−11%), 42 days in S3 (−26%) and 30 days in S4 (−47%), effectively halving the time that the plant spent idle (Fig. 4c).

At a subdaily timescale, the addition of solar practically eliminates shutdowns in the hours after noon (Fig. 4c, insets). Subsequently adding wind power reduces shutdowns throughout the day. Despite this overall outcome, S2 and S3 have, on average, more plant shutdowns concentrated in certain time blocks than S1. This is because, in normal years, the hybridization with VREs allows the Complejo Paute to aim to meet a higher target production than without VREs. As the model is myopic for 2024, the operational schemes in each scenario strive to meet the corresponding peak loads even during the drought period. The effect is a higher strain on the reservoirs during ‘off-solar’ hours vis-à-vis S1. However, this is compensated by the much reduced strain during the ‘solarized’ hours, and the effect is largely absent in S4 owing to improved exploitation of diurnal and seasonal hydro–VRE synergies.

## Towards a resilient Ecuadorian power system

Although the alleviated impact of droughts on reservoir operation is encouraging, none of the VRE scenarios would have safeguarded the Paute Molino plant from shutdowns covering almost half of November 2024, the worst period of the crisis (Fig. 4c). We therefore investigated an additional measure: more prudent overall reservoir management that deliberately keeps more water stored in the reservoirs during normal years (Methods). This would raise water levels overall so that future crisis periods are entered under better conditions and can be better traversed even when unforeseen (Supplementary Note 6). The downside is that part of normal-year hydropower generation may be foregone and more water spilled, additionally resulting in diminished potential for VRE integration.

**Fig. 5:**
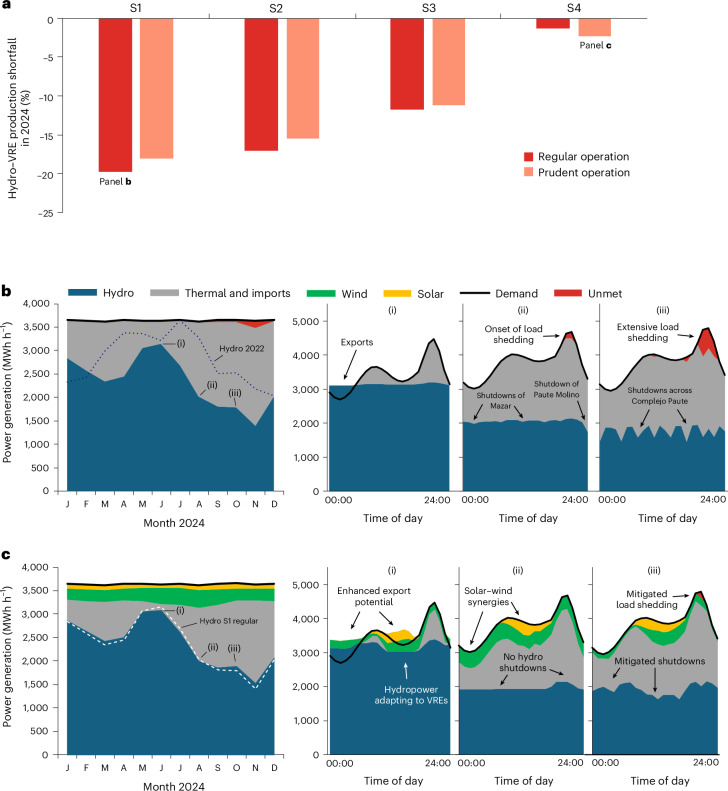
Ecuador’s power generation shortfalls in 2024. **a**, Overall power generation shortfall across Ecuador, defined as the generation from hydropower and VREs in 2024 compared with annual average hydropower generation in the calibration period, under S1–S4, each simulated with a regular operational scheme and a prudent operational scheme. **b**,**c**, Seasonal (2024) and hourly (3 days in 2024) power mix of Ecuador under S1 regular operation (**b**) and S4 prudent operation (**c**): 29 June (i), 7 August (ii) and 8 October (iii). For comparison purposes, the seasonal hydropower generation curve from 2022 has been added to **b**, and the curve from S1 (regular) to **c**. Note that the category ‘Thermal and imports’ assumes full availability of thermal and import capacity (as of 2024) when needed. The category ‘Unmet’ represents moments when this capacity would not have been sufficient to close the gap between hydro–VRE generation and demand (see Fig. 6 for further analysis). Hourly demand curves are taken from ref. ^87^.

Figure 5a shows Ecuador’s power generation shortfall during the crisis (compared with normal years, see Fig. 3c) after accounting for VRE integration into the Complejo Paute and for power generation from Ecuador’s other hydro plants (Fig. 1, Methods and Supplementary Note 2). The outcomes of scenarios S1–S4 are compared under both regular operation (as in Fig. 4) and prudent operation, the latter being defined as the first operational scheme achieving zero shutdowns of the Paute Molino plant across 2024 when gradually introducing more prudency. Under all scenarios, this prudent scheme comes down to deliberately foregoing about 2% of the average hydropower generation of the Complejo Paute in normal years, corresponding to a reduction in the capacity factor of the Complejo Paute of around one percentage point, but in return being able to generate around 7% more electricity in the crisis year compared with regular operation. This difference between regular and prudent operation is important under S1 for alleviating the Ecuador-wide shortfall under 2024 conditions, but loses relative weight under S2–S4 as the contribution of VRE generation in compensating the shortfall becomes dominant. Each successive VRE integration scenario bridges the shortfall better, and under S4 it is nearly reduced to zero. In all cases, the VREs are more than sufficient to compensate the small amounts of hydropower foregone in normal years. Correspondingly, the additional expenses on fossil fuels that prudent reservoir operation would cause in normal years without VRE integration (S1) can be more than offset by the fossil fuel savings brought about by VRE integration (S2–S4). In S4, the additional annual expenses on fuel due to prudent operation are only 5–10% of the annual fuel savings that VRE integration brings.

Figure 5b,c shows simulations of Ecuador’s power mix in 2024 for two scenarios—S1 under regular operation (Fig. 5b) and S4 under prudent operation (Fig. 5c)—at the seasonal level and for three example days during different stages of the crisis (Methods). Figure 5b shows that during the ‘least critical’ months (May–July 2024), Ecuador was even able to export small quantities of electricity, a finding confirmed by official statistics^51^. Later in the year, intermittent shutdowns of Mazar and Paute Molino had to be introduced, a situation that considerably worsened in October–November and constantly affected the entire Complejo Paute (as also seen in observational data^52^; Supplementary Note 7), leading to substantial unmet demand (load shedding) due to insufficient thermal capacity to compensate the hydropower deficit. The large-scale introduction of VREs under S4 and the corresponding exploitation of hydro–solar–wind synergies would not only have increased the export potential during the least critical months (Fig. 5c and Supplementary Note 8) but also substantially mitigated the onset, duration and dynamics of shutdowns. Prudent operation would have fully prevented the shutdowns of Paute Molino, leaving only less frequent shutdowns of Mazar (which requires a higher minimum turbine load to run than Paute Molino) in the worst crisis months. Together, these measures would have drastically reduced the amount of unmet demand. Across these scenarios, VRE integration logically increases hydropower ramping needs, whereas prudent management decreases them; overall, these needs remain well within feasible limits. For thermal plants and imports, VRE integration has only a minor impact on average ramping needs as hydropower delivers most of the additional flexibility needed. A detailed discussion is provided in Supplementary Note 9.

**Fig. 6:**
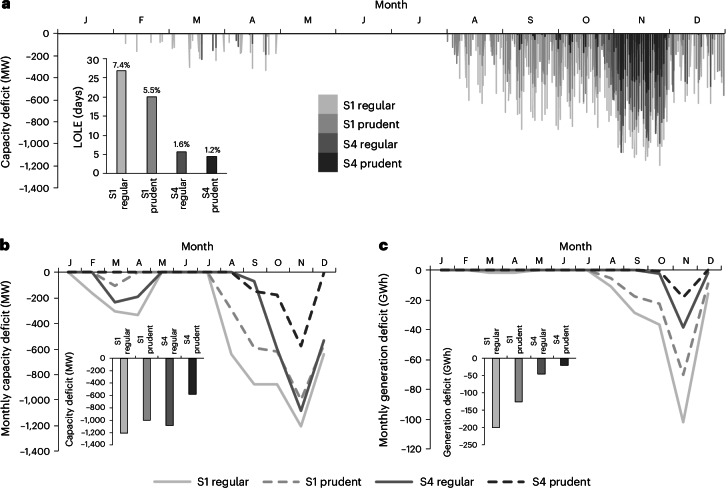
Ecuador’s capacity and generation deficit in 2024. **a**, Capacity deficit at the hourly scale for regular and prudent operation of both scenarios S1 and S4. Inset: the corresponding LOLE under the same conditions. The percentages indicate the annual share of LOLE days. **b**,**c**, The corresponding maximum capacity deficit (**b**) and total generation deficit (**c**) for each month. Insets: the corresponding data for the entire year.

Figure 6a shows a time series of the capacity deficit for each hour of 2024, comparing S1 and S4 under both operational variants. This capacity deficit is defined as the instantaneous gap between the residual power demand to be met by thermal plants and imports on one hand and the thermal and import capacity that Ecuador actually had available in 2024 on the other (that is, corresponding to the ‘unmet’ time series in Fig. 5b,c). Clearly, both hydro–VRE hybridization and prudent reservoir operation would have shortened the amount of time in which the system had insufficient capacity to meet demand, also known as the loss of load expectation (LOLE). Of the two options, VRE hybridization has the larger effect in reducing LOLE (Fig. 6a, inset), which drops from 27 days (S1 under regular operation) to 4 days (S4 under prudent operation). Furthermore, aggregated monthly capacity (Fig. 6b) and generation (Fig. 6c) deficits can be derived. Both quantities are important to estimate the remaining investment needs to make the system ‘2024-proof’ in each scenario, with the capacity deficit determining the needed capital expenses (costs per megawatt) and the generation deficit the costs of fuel or imports (costs per gigawatt hour). Our results align with reported statistics: we found a capacity deficit of 1,210 MW under the reference scenario in late 2024 (Fig. 6b), close to the 1,298 MW quoted by official sources^53^.

To reduce the capacity deficit, prudent reservoir management turns out to be a more effective strategy than VRE hybridization, but a combination of both is substantially better than either separately (Fig. 6b and Supplementary Note 10). To reduce the generation deficit, VRE hybridization works better than prudent operation, but the combination again far outperforms the individual strategies (Fig. 6c). While November 2024 remains, under all scenarios, the most problematic month, combining VRE hybridization with prudent management reduces (compared with the reference scenario, S1 regular) the capacity deficit from 1,210 MW to 580 MW (−52%) and the generation deficit from 200 GWh to 20 GWh (−90%). These deficits could be covered by, for example, operationalizing the planned 1,000-MW interconnector with Peru^54^, which suffered no energy crisis^55^.

Notably, the ratio of the capacity deficit reduction to the corresponding hybridizable VRE capacity is quite high: we found 59% for the case presented in Fig. 6 and a range of 50–65% across various sensitivity studies on model resolution (Supplementary Note 11), with the amount of hybridizable VREs always around 1 GW. In other words, the addition of gigawatt-scale VRE capacity, alongside prudent operation, reduces the additional peaking needs by an amount equivalent to one-half to two-thirds of the VRE capacity. That ratio could be interpreted as an effective ‘capacity credit’ of hydro-supported VREs, defined as the percentage of a resource’s installed capacity that can be relied upon during periods of peak demand and/or highest system stress. For VRE sources, whose capacity credit has traditionally been considered (close to) zero due to their non-dispatchable character^56^, such relatively high capacity credit values would be promising indeed. However, note that the effect is largely an indirect one: it is not that VREs are guaranteed to be always available in the precise moments of system stress, but rather that the hydro–VRE synergies alleviate that stress by positively impacting hydropower reservoir operation in critical periods (Supplementary Note 10). In the case of the 2024 crisis in Ecuador, this is because the seasonal period with the highest VRE potential would have coincided well with the period that manifested the highest river discharge shortfall, so hydro–VRE hybridization would have allowed more water to have been kept in the reservoirs during those months, enabling increased hydropower generation later in the year. Thus, it is best to think of this ratio as a ‘virtual’ or ‘indirect’ capacity credit of VREs, requiring appropriate reservoir operation to manifest.

## Conclusion and discussion

Ecuador could diversify its electricity mix by using its flexible hydropower capacity, mostly concentrated in the Complejo Paute, to support the grid integration of VREs. This, alongside moderately more prudent reservoir operation, would have largely safeguarded Ecuador from the impacts of the 2023–2024 drought on the country’s hydro-dominated electricity system, mitigating the experienced loss of economic value and human well-being. In particular, it would have (1) more than halved the need for additional peak capacity for thermal power (or imports), (2) drastically reduced the need for additional fuel consumption (or imports) and (3) largely avoided the stop–start profiles of hydropower on the Paute River during the crisis.

In 2024, Ecuador ended up producing 33% more electricity from thermal plants than in 2023, and 90% more than in 2022, as it attempted to cover the shortfall left by the hydropower crisis with fossil fuels^57^. Nevertheless, the available thermal capacity was insufficient to adequately compensate for the loss of hydropower and the country could not procure enough fuel to run its thermal plants at full capacity^58^. In the second half of 2024, Ecuador had so few options available to fill its capacity and generation deficit that it resorted to leasing a powership barge in an attempt to mitigate the daily, countrywide blackouts. Against this backdrop, the long-term safeguards that VREs could introduce into Ecuador’s power system may provide compelling reasons for diversification: while VREs are generally considered to have low capacity credit^56^ and not usually seen as obvious candidates for bridging capacity deficits, our study shows that hydro-supported VRE integration, under the right circumstances, allows the exploitation of ‘hidden’ synergies between renewables that can substantially improve prospects to guide electricity systems through extreme periods. These prospects can be linked to a newly identified ‘virtual’ or ‘indirect’ capacity credit that VREs, under the right circumstances, may have in drought-stressed systems.

Of course, hydro–VRE hybridization is only one among various possible resilience measures for Ecuador. Other candidates include further expanding the fossil fuel-powered fleet, building more hydropower plants on the Pacific side to better exploit the Amazonian–Pacific complementarity, constructing more interconnectors to link neighbouring Colombia and Peru, and deploying storage technologies. These measures can, and most likely will, coexist. On the fossil fuel option, we estimate that VREs may well be cheaper than new fossil-based power in the Ecuadorian context, even under pessimistic assumptions (Supplementary Note 12). The VRE option also avoids the costs of ‘emergency’ procurement of fossil fuels during crises and the associated supply chain bottlenecks^58^. The further expansion of the hydropower fleet is already underway, as evidenced, for example, by the inauguration of the 254-MW Toachi Pilatón plant on the Pacific side in early 2025 (Fig. 1). Although the 2024 drought largely suppressed the Amazonian–Pacific complementarity, a more balanced hydropower portfolio on both drainage sides may facilitate more prudent reservoir management in normal years, increasing resilience to dry periods. Concerning transmission, interconnectors are more likely to complement hydro–VRE hybridization than compete with it^7,16,24,59^. If Ecuador built out its VRE fleet and simultaneously advocated for more interconnections, it could gain opportunities to export excess VREs during part of the year (Supplementary Note 8) while increasing its prospects of resilience during dry periods. Regarding storage, battery and pumped hydro schemes could support further VRE integration, but these typically offer day–night storage and would be insufficient to overcome the months-long capacity and generation deficit periods that the drought of 2024 created. Seasonal storage through power-to-X technologies could be a conceivable option, for instance, by producing green hydrogen from excess VREs and storing it for later combustion in hydrogen-fired power plants. However, this option is clearly more expensive than our proposed strategy, which uses solar and wind power directly as a fortification mechanism instead of requiring investment in electrolysers, hydrogen-fired power plants and hydrogen storage units in addition to the VREs.

Overall, the best way to identify optimal combinations of technologies for Ecuador’s power system would be through long-term cost-optimization tools. However, such tools mostly do not allow the explicit modelling of reservoir management, and when they do, the computational cost tends to prohibit full hourly runs spanning multiple years^19^. In addition, such cost-optimization tools are usually not myopic on extremes as they optimize over larger time horizons. This makes them less appropriate for the analysis of drought-induced crises compared with our approach as they would be relatively ill-suited to, for example, identify the diurnal, seasonal and multiannual effects of the identified hydro–VRE synergies on reservoir operation. Thus, we see our study as a potential complement and/or input to long-term cost-optimization modelling, which, in the context of system resilience, could be used for more comprehensive adequacy assessments, for example, through stochastic consideration of unforeseen VRE fluctuations, demand uncertainties, unplanned power plant outages and transmission line failures, thus accounting for uncertainty in optimization models that are principally deterministic^60,61^. By running a large ensemble of optimization runs, this would give a range of possible system outcomes, allowing to plan for sufficient capacity to ensure resilience not only to droughts but also against various types of compound events^62,63^. An important avenue for future research would be to explore how best to iteratively couple the REVUB approach with such optimization tools.

To pursue the suggested gigawatt-scale VRE build-out in Ecuador, various other challenges will have to be faced. As the infrastructure for VRE deployment requires time to build not only power plants but also transmission lines, in Ecuador’s case in highly mountainous terrain, it is recommended that the opportunities and barriers for further VRE build-out in Ecuador be urgently studied. Here, too, cost-optimization models could play a role, for example, to identify optimal VRE plant placements considering grid expansion costs, as in ref. ^64^. Policy and regulatory incentivization may also prove valuable to promote VRE expansion. The effective subsidization of hydropower and fossil fuels in Ecuador currently still hinders the cost-competitiveness of VREs^29^. The country also lacks a systematic policy framework to attract VRE investment^29,65^ and many of the projects targeted by Ecuador’s 2023–2032 Electricity Masterplan^66^, including hundreds of megawatts of auctioned VREs^67^, have not (yet) been realized^65^. Regarding market measures, Ecuador currently has no legal framework for the remuneration of supply flexibility through, for example, ancillary services markets that could provide incentives to hydropower plant operators (and other flexibility providers) to adapt operations to support VRE uptake^65,68^. Nevertheless, care must be taken when designing new measures to ensure generation adequacy during scarcity in the context of hydro–VRE-heavy systems. For instance, in neighbouring Colombia, it has been found that the implementation of ‘reliability options’ (financial contracts meant to induce power producers to be available during times of scarcity) sometimes creates perverse incentives for less prudent operation of hydropower plants^69^. Financing modalities are also crucial: VRE projects in Ecuador are reported to have among Latin America’s highest capital costs, at more than 10%, potentially a strong deterrent for investors^70^. Fortunately, Ecuador is not completely a VRE greenfield: it already has about 74 MW of wind power capacity. The scenarios examined here, however, would require a VRE fleet that is around ten times larger.

In our view, the main uncertainties and limitations of our study, impacting their potential applicability to future power sector planning in Ecuador, are threefold. First, reservoir operation is more complex than running model simulations and extracting ‘optimal’ dispatch strategies; in reality, many ad hoc decisions in reservoir management have to be taken on the basis of unexpected surges or drops in power demand, unforeseen behaviour of other power plants, unforeseen changes in available transmission capacity, the need for sedimentation reduction and other factors. These may impact the feasibility of consistently harmonized hydro–VRE hybridization. Second, there exists uncertainty around potential changes in hourly and seasonal load profiles in the future and their interannual variability, as influenced by economic development and climate change. This may have ramifications for the precise interplay between hydro–VRE supply and electricity demand. Third, climate change itself may impact renewable resource potential, chiefly for hydropower, which may be affected by various basin-level trends in river discharge (for example, higher/lower average flow and increases in extremes), thus also affecting the hydro–VRE hybridization potential.

Research shows that anthropogenic climate change made the 2023–2024 drought in the Amazon basin 10–30 times more likely^71^. With El Niño extremes projected to increase in frequency^72,73^, extreme droughts would evolve from a 1-in-100-year occurrence today to a 1-in-30-year occurrence in a 2 °C warmer world^71,74^. Safeguards against droughts will thus remain of crucial importance for Ecuador, and as various other nations in Central and South America, as well as in Africa and Asia, are similarly hydro-dependent, our findings may inspire further country-level analysis on resilience planning in and outside the region^73^.

## Methods

### REVUB model implementation

To model hydropower operation and dispatch under different levels of VRE hybridization, we used the REVUB model. The model software has been used in various studies to analyse hydro–VRE hybridization in a range of contexts, notably for smart renewable energy targets across West Africa^7^, aligned water–energy planning in Ghana and Burkina Faso^49^, enabling cross-border energy and water cooperation between Ethiopia, Sudan and Egypt^16^, foregoing future hydropower plant investment in favour of solar photovoltaics in Guinée^47^ and displacing thermal power from Suriname’s grid through enabling a climate-resilient hydro–VRE mix^10,46,48^. The present study represents the REVUB model’s first application to Ecuador and its first application centred around extreme drought conditions.

The equations and modelling principles of REVUB are explained in its user manual^75^ and summarized briefly here. In essence, the model serves to derive—at hourly resolution across multiannual periods—bespoke hydropower plant-level operation rules adapted to meet appropriate target loads in conjunction with VRE sources while respecting minimum turbine load and minimum environmental flow needs, constrained by technical and hydrological limitations on turbine- and reservoir-level flexibility, as well as by the requirement to keep water levels within operational ranges and the need to coordinate between different reservoirs in a cascade.

To derive these operational rules, REVUB automatically performs a range of plant-level simulations covering an ensemble of target load levels, running through the ensemble from low to high loads. As the model iterates through ever higher target loads, it must hybridize more and more VREs with the hydropower operation to remain able to meet the target load, and keep adapting the hydropower operation accordingly. In each run, the model starts from the same initial reservoir storage state and marches forwards in time, adapting the amount of turbined water to meet that run’s residual target load for each time step after accounting for the VRE contribution and recalculating the reservoir state at each following time step.

For each ensemble member, REVUB verifies ex post to what extent reservoir-level outcomes are aligned with idealized ‘rule curves’ based on a calibration period chosen by the user. These rule curves represent seasonal drawdown/refill curves according to parameterized, logarithmic–exponential release rules for reservoir outflow^75^ and take the average river discharge across the calibration period as input. (The choice of calibration period is thus important as it determines how optimistic/pessimistic the rule curves are; usually, for realistic simulations, the chosen calibration period should be representative of historical conditions.) The model then selects as the optimal solution the outcome in which those reservoir-level criteria are best respected (that is, where the target load is neither so low that it would underexploit the hydropower flexibility nor so high that it would unduly strain reservoir operation). That optimal solution, which integrates short-term balancing and flexibility needs with long-term operational adequacy, is associated with a certain optimal target load and optimal VRE capacity, which can be considered ‘hybridizable’ with the investigated hydropower plant.

Cascaded hydropower systems are modelled by REVUB in two possible ways, with either the upstream plant or the downstream plant serving as the ‘leading’ unit, which is the main determinant of cascade operation. The leading unit is always simulated first and co-determines the operation of the other ‘lagging’ unit. If the downstream plant is the leading unit (for example, by virtue of having a higher installed capacity and/or more flexibility owing to a higher number of turbines), it is assumed that the operation of the downstream plant will determine the drawdown of both reservoirs. REVUB models the joint operation of two such cascaded reservoirs on the assumption that any change in cumulatively stored volume across the reservoirs is divided over both according to a given share, that is, ‘harmonized’ operation in which reservoirs usually have synchronized refilling and drawdown. Here, we took this share to be equal to each reservoir’s share in maximum total volume. Once the simulation for the leading unit, drawing upon both reservoirs’ storage capacity, is completed, the model simulates the operation of the lagging unit, now constrained by the needs of the leading unit for cascade reservoir drawdown. This is done by replacing the lagging unit’s regular ‘rule curve’ (see above) with the implied refill/drawdown curve necessary for the operation of the leading unit. If, instead, the upstream plant is the leading unit (for example, because the downstream plant has limited to no storage capacity), the REVUB model uses the simulated outflow of the upstream plant to obtain the inflow of the downstream unit. (Details on cascade modelling and its limitations in REVUB are given in Note 7 of the REVUB user manual^75^.)

The REVUB model takes various technical hydropower plant data as input (such as rated capacity, bathymetry curves, number of turbines, turbine efficiency, design discharge, ramp rate and critical lake levels), as well as time series describing the hydrological conditions, solar and wind power generation curves, and demand variability, plus several operational parameters, such as the minimum stable load of the hydropower plant as determined by its turbine characteristics or the fraction of inflow effectively used for drawdown and refill purposes. The principal data sources are discussed below and we provide a full, tabulated list of input parameters, as well as their values under all scenarios, in the REVUB input files available via Zenodo^76^. Those input files can also be used to replicate this study’s scenarios.

The REVUB model can run any scenario of the present study at full hourly resolution in a few minutes on a standard laptop computer.

### Hydropower data for the Complejo Paute

For the three plants in the Complejo Paute, monthly river flow data for the modelling period 2011–2024 were obtained from the CELEC SUR data portal^52^ (noting that the Sopladora plant only started operation in 2016). Net evaporation losses from the surfaces of the reservoirs in the Complejo Paute were estimated to be a second-order effect and were not taken into account given that the Mazar and Amaluza dams impound relatively narrow valleys, resulting in very low values of inundated surface area per unit capacity (0.65 ha MW^−1^ for the Complejo Paute) and correspondingly low water footprints (see ref. ^77^ for a comparison of this indicator across hydropower plants on multiple continents). Technical hydropower plant data (see ‘REVUB model implementation’ above) were taken from a range of sources for each individual hydropower plant and are documented and listed in the REVUB model input file provided on Zenodo^76^. In the cascade system (with Paute Molino receiving Mazar outflow plus some lateral inflow into Lake Amaluza^52,57^ and Sopladora receiving Paute Molino outflow through a discharge tunnel^78^), Paute Molino was defined as the leading unit in both cases (see ‘REVUB model implementation’ above and also Supplementary Note 2).

The REVUB model was calibrated with observational data by comparing time series data of monthly lake levels (Fig. 3b) and annual electricity generation (Fig. 3c) between model and measurements. The corresponding calibration parameters in REVUB were (1) the regulation fraction *f*_reg_ determining which share of the river discharge is allocated for regulated use (to calibrate lake level fluctuations) and (2) the turbine efficiency parameter *η* (to calibrate power generation statistics). Further details on these parameters are given in the REVUB user manual^75^. The calibration of both parameters leads not only to an encouraging match between model and observations for the lake level and power generation time series (Fig. 3b,c) but also for the length of plant shutdowns in the Complejo Paute in 2024 (Fig. 3d). Full details of the calibration are provided in Supplementary Note 2.

We show in Supplementary Note 11 that the conclusions of this study remain unchanged when running alternative simulations using daily, instead of monthly, averages for Paute River flow, keeping all other things equal. However, daily flow data were available only for the Complejo Paute and the Minas San Francisco plant (plants (i)–(iv) in Fig. 1). For the other hydropower plants (v)–(ix), monthly resolution at most could be obtained and certain years were missing in the time series (see ‘Full power mix simulation’ below). Thus, we believe it is instructive to have a scenario with all input discharge data at monthly resolution so as to make it clear that the analysis also yields useful results at this resolution.

### Solar and wind power data

Geospatially resolved solar and wind power generation curves were based on the open-source Model Supply Regions (MSR) methodology developed originally by the International Renewable Energy Agency (IRENA) for Africa^79^ and later replicated for Central and South America by the authors of this study^80^. The methodology combines the kilometre-scale spatial resolution of the Global Solar Atlas^81^ and Global Wind Atlas^82^ with the hour-scale temporal resolution of the meteorological data available in the ERA5 reanalysis dataset^83^. MSR datasets include hourly capacity factor curves for the most attractive locations for solar and wind power plant development within each country’s territory, where the recommended criterion for attractiveness is the estimated plant-level levelized cost of electricity (LCOE). In the MSR methodology, the capital expenses of necessary grid expansion, depending on the remoteness of the resource, are included in this LCOE calculation (see also Supplementary Note 12). Areas deemed unsuitable due to adverse terrain, slope, conflicting land use, presence of natural reserves or prohibited locations, high population densities and other factors (described in ref. ^79^) are excluded. Thus, the dataset gives the individual contiguous areas, ranked by LCOE from low to high, that could house solar or wind power plants at kilometre-scale resolution and with hour-scale generation curves for each.

Here, we started from the dataset in ref. ^80^, creating a screened selection of attractive areas by taking the best-ranked ones (in LCOE terms) up to a coverage of 5% of Ecuador’s territory and then a subselection of candidates for hybridization with hydropower by taking all areas falling within a 200-km radius of the Complejo Paute (Supplementary Note 3). As the LCOE includes grid expansion costs, this automatically excludes locations with prohibitive transmission build-out requirements. In the case of wind power, the identified locations match closely the sites where Ecuador has already built utility-scale wind farms: the sites of both the 16.5-MW Villonaco project and the 50-MW Minas de Huascachaca project are part of the subselection. (For solar power, such a comparison cannot yet be performed due to the absence of large-scale solar farms in the country, but the clusters match well CELEC’s analysis of solar photovoltaic potential in Ecuador^84^.)

Given Ecuador’s high average elevation, the probable additional costs in transporting, installing and maintaining wind turbines at high altitudes must be taken into account, as well as potential special turbine design requirements^85^. While the Villonaco wind farm (which entered into service in 2013) is located at about 2,650 m.a.s.l., the Minas de Huascachaca project (from 2023) lies at the much lower altitude of 1,100 m.a.s.l. We chose here to make a further refined subselection for wind power and focused only on the locations below 2,000 m.a.s.l. as we assumed that, in the case that Ecuador opts for the kind of fast and extensive deployment of VREs suggested here, it may prefer to exploit lower-lying high-potential locations first. However, we ran an additional scenario including all wind locations below 3,000 m.a.s.l. as a sensitivity check (see ‘Scenario design’ below).

The VRE profile envelopes in Fig. 2d represent weighted averages of site-specific capacity factors across the aforementioned subselections for the full period of 2011–2024, with lower and upper bounds given by monthly minima and maxima. To make the solar and wind envelopes easily comparable (as the average capacity factor is ~19% for solar and ~54% for wind), both envelopes were normalized by the respective resource’s average capacity factor across the entire period of 2011–2024.

For the results shown in Figs. 4–6, we used a single year’s meteorological data for VREs throughout the modelling period for demonstration purposes. The reason for this was that the interannual variability and spread in seasonal variability of VREs are vastly inferior to that of river flow, and VRE yield is practically independent of drought occurrence. Thus, for the purposes of investigating hydro–VRE hybridization, the interannual variability of VRE yield plays an insubstantial role compared with that of hydropower (details and visualizations are given in Supplementary Note 11). Here we chose the meteorological year 2018, which appears to have been one of the most ‘average’ years in the investigated period: it was the year in which both Paute River discharge and yield at the Villonaco wind site were closest to their average across the modelling period^52,57,86^. We show in Supplementary Note 11 that the conclusions of the study remain unchanged when using the full 2011–2024 time period for hourly VRE yield instead of a single weather year.

### Demand variability

To represent the hourly-to-seasonal variabilities in electricity demand profiles, we used the Ecuador demand curve from the dataset provided in ref. ^87^ containing synthetic demand profiles for all countries in the world obtained using a model that decomposes load profiles into a sum of harmonic functions at various timescales (hourly, daily, weekly and seasonal). The model in ref. ^87^ considers a range of explanatory variables, including peak-to-average demand, temperature regimes, gross domestic product, population size, industrial production and day duration, and was calibrated to the real load profiles of several dozen countries across all continents. The set of calibration countries did not include Ecuador, but neighbouring Colombia was included (as were Mexico, Argentina, Chile and Brazil, the other Latin America countries to be included).

In our REVUB simulations, it was assumed that the target load that the hydro–VRE mix should meet has the same normalized shape as the profile extracted from ref. ^87^. The target load curve thus equals this normalized hourly profile multiplied by a certain average target load level expressed in megawatts; as explained above, for each scenario, the model cycles through an ensemble of low-to-high target load levels to identify the optimal level that the hydro–VRE configuration of each scenario can meet while respecting all constraints in that scenario. The REVUB results thus show how hydropower would need to be operated such that the hydro–VRE mix performs load-following for a certain fraction of Ecuadorian hourly power demand; the remainder of that power demand was then assumed to be met by thermal power or imports, insofar as capacity is available (see Figs. 5 and 6 and ‘Full power mix simulation’ below).

While there is uncertainty in using a synthetic hourly load shape from the literature, in our view, there was no credible alternative. Even if we had had access to the hourly load profile from 2024 from the grid operator, that load data would already have included the blackouts and load shedding events implemented by the Ecuadorian government (an extreme form of demand response) to cope with the drought and thus would not have been usable for the investigation.

### Scenario design

The presented scenarios S1–S4, as well as a further scenario S5, can be summarized as follows:

- S1 represents the reference scenario, in which the Complejo Paute is operated flexibly to follow target loads, without considering VRE integration.
- In S2, the Complejo Paute operation is hybridized with solar power. The production curve of solar represents the weighted average of production profiles across the identified subselection of solar power sites from the MSR analysis (see ‘Solar and wind power data’ above).
- In S3, the Complejo Paute operation is hybridized with a mix of solar and wind power. The capacity mix of solar and wind is fixed at 50% solar and 50% wind, meaning that each megawatt of solar power in the hybridized mix is matched with 1 MW of wind power. (This 50:50 split is intended to demonstrate the different effects of solar and wind integration, not as an ‘ideal’ mix. A cost-optimal ratio of solar and wind power would be determined through long-term cost-optimization tools; see also the Conclusion and discussion.) We used the identified subselection of wind power sites below 2,000 m.a.s.l. (see ‘Solar and wind power data’ above).
- S4 is the same as S3, except that the simulation constraint limiting overproduction of the hydro–VRE mix (compared with the target load level) is substantially relaxed (from being allowed 10% of the time to being allowed 35% of the time, inspired by ref. ^16^). This allows more VREs to be hybridized with hydropower by accepting higher levels of instantaneous VRE overproduction compared with the hydro–VRE target load.
- For a sensitivity analysis, one further scenario (S5) is defined, which is discussed in Supplementary Note 13: S5 is the same as S4, except that the wind power site subselection is extended to cover suitable locations with a maximum elevation of 3,000 m.a.s.l.

The precise simulation settings for each scenario can be found in the REVUB model input file available via Zenodo^76^.

### Modelling prudent reservoir operation

Prudent reservoir operation was modelled as follows: after having identified the optimal hydro–VRE hybridization solution for each scenario (see ‘REVUB model implementation’ above), referred to here as regular operation, we re-ran the same scenario for a ‘reverse ensemble’ of target load levels, starting just below the optimal load level identified for regular operation and incrementally reducing it. With a marginally lower load to meet in each reverse ensemble member, the strain on the hydropower plant is eased progressively, raising average lake levels across the modelling period and reducing the probability of plant shutdowns occurring in the crisis period. The ‘prudent scenario’ shown in our analysis corresponds to the first solution in the reverse ensemble in which the total duration of shutdowns of the Paute Molino plant in 2024 reaches zero.

Introducing prudency into the operation tends to have a slight negative effect on hydropower generation in normal years as less water is turbined (the water balance being closed by the reservoir having less buffer capacity in the wet times of the year due to overall higher water levels, thus leading to the release of more water through spillways; Supplementary Note 6). Accordingly, the capacity for supporting VREs tends to be somewhat diminished too. However, there are exceptions: in some cases, the increase in the hydraulic head resulting from the increased average water levels can compensate for this foregone turbined water. In our simulations, this was the case for Mazar, whose generation of hydropower was nearly invariant between regular and prudent operation.

### Full power mix simulation

We also ran REVUB simulations of Ecuador’s principal hydropower plants outside the Complejo Paute (those shown in Fig. 1) to perform the analysis presented in Figs. 5 and 6. Those hydropower plants were modelled as run-of-river schemes due to minimal or absent storage, except for the Marcel Laniado plant (vi), whose dam impounds the 6,000 million m^3^ Daule Peripa reservoir; it was assumed that Marcel Laniado would behave according to S1. The other (run-of-river) plants were modelled assuming that all incoming inflow is immediately turbined, with an upper cap on production set by the design discharge of the turbines^75^. The Toachi Pilatón plant (x) was not modelled as it only became operational in 2025, after the crisis.

For the Minas San Francisco plant (iv), monthly river discharge data were obtained from the plant operator CELEC^52^; for all other hydropower plants outside the Complejo Paute, the data were taken from the annual reports of the grid operator CENACE^57^. In some individual years, for certain hydropower plants, these annual reports only provided the yearly average river discharge for that year instead of the monthly variability, in which case the average monthly variability across all remaining annual reports was bias-corrected to the reported yearly average river discharge for the year with missing monthly data to obtain a proxy time series at monthly resolution for that year. For all hydropower plants, the turbine efficiency parameter *η* was used to calibrate REVUB outcomes to average power generation statistics from the same CELEC^52^ and CENACE^57^ sources (full results of the calibration for all simulated hydropower plants are provided in Supplementary Note 2).

In Fig. 5b,c, ‘hydropower’ represents the sum of the production of the nine hydro plants (i)–(ix) explicitly simulated with REVUB under each scenario, plus a correction term to account for the fact that the power generation of those nine plants, according to the 2024 CENACE annual report, represented slightly above 80% of the production of Ecuador’s full hydropower fleet in that year^51^ (the remaining 20% coming from 48 further hydropower plants, most of which have installed capacities of ≤20 MW). This correction term corresponds to the summed hydropower time series of plants (i)–(ix) under the ‘S1 regular’ reference scenario multiplied by a correction factor of 20% and subsequently averaged at a monthly timescale. Thus, the assumption is that those remaining 48 hydropower plants (1) have the same aggregated seasonal profile as plants (i)–(ix), (2) behave identically under all scenarios and (3) do not contribute to flexibility provision and VRE hybridization. In the same figure, VREs represent the sum of the VREs hybridized with the Complejo Paute as per the REVUB simulations.

Using the REVUB simulation output, the needs for thermal power and imports in 2024 were then calculated ex post at the hourly level as the difference between the demand curve and the sum of hydro and VRE generation, with the demand curve representing the product of the normalized load profile and the annual average load according to the grid operator^51^ (see ‘Demand variability’ above). Whenever the sum of hydro and VRE generation exceeded the demand curve, the need for thermal power and imports was defined as zero and the difference was interpreted instead as the potential to export electricity, as indicated in Fig. 5b(i),c(i) (see also Supplementary Note 8). Whenever the needs for thermal power and imports exceeded the capacity that Ecuador had available in 2024, the difference was defined as unmet demand (see ‘Identifying capacity and generation deficits’ below).

### Identifying capacity and generation deficits

The unmet demand in Fig. 5b,c and the capacity and generation deficits in Fig. 6 were calculated by comparing the hourly needs for thermal generation and imports (see ‘Full power mix simulation’ above) with the thermal and import capacity that Ecuador had at its disposal in 2024. Here, this was taken to be 1,862 MW of thermal capacity^88^, 450 MW of interconnection capacity with Colombia^89^ and 80 MW with Peru^90^. Whenever the needs exceeded the sum of those capacities in a given hour, that hour was registered in the LOLE count, and the difference between needs and capacities for that hour was registered as a capacity deficit (in megawatts) and as a generation deficit (in megawatt hours). The full hourly time series of this difference is shown in Fig. 6a.

The aggregated capacity deficit at the monthly (annual) scale was calculated as the maximum of the hourly capacity deficit during that month (year), while the aggregated generation deficit at the monthly (annual) scale was calculated as the sum of the hourly generation deficit during that month (year). In reality, the generation deficit would probably have turned out to be larger than in our calculation because thermal plants and import lines are not guaranteed to be available 100% of the time when there is a capacity deficit, nor necessarily at full capacity.

### Reporting summary

Further information on research design is available in the Nature Portfolio Reporting Summary linked to this article.

## Supplementary information

Supplementary Information Supplementary Notes 1–13, Figs. 1–17 and Tables 1–3.

Reporting Summary

## Data Availability

The MSR workflow is available via GitHub at https://github.com/SPLATteam/Model-Supply-Regions-MSR-Toolset. The high-resolution hydrological and energy-related data for CELEC Sur are available in ref. ^52^. Hydrological and energy-related data from CENACE are available in ref. ^57^. The REVUB model input files used to run this study’s scenarios and supplementary data sources are available via Zenodo at 10.5281/zenodo.15854447 (ref. ^76^).

## Code availability

The open-source Python-based REVUB model (v.1.1) is freely available via Github at https://github.com/VUB-HYDR/REVUB.
