# Supplementary material for: Variable renewables fortify Ecuador’s power system against recurrences of drought-driven energy crises
Source: Nat Water. 2026 Apr 7;4(5):571–85. doi: 10.1038/s44221-026-00617-w (PMC13197224; doi:10.1038/s44221-026-00617-w)
Supplement: Supplementary file 1 — Supplementary Notes 1–13, Figs. 1–17 and Tables 1–3. [file 44221_2026_617_MOESM1_ESM.pdf]

nature water

Supplementary information https://doi.org/10.1038/s44221-026-00617-w

Variable renewables fortify Ecuador's power system against recurrences of drought-driven energy crises

In the format provided by the authors and unedited

Contents

Supplementary Note 1	Robustness of extreme-year synergy	2
Supplementary Note 2	REVUB model calibration and validation	3
Supplementary Note 3	Geospatial representation of VRE	7
Supplementary Note 4	Solar-driven "micro-recoveries"	9
Supplementary Note 5	Solar-wind synergies	10
Supplementary Note 6	Regular versus prudent reservoir operation	11
Supplementary Note 7	Stop-restart hydropower cycles during the crisis peak	12
Supplementary Note 8	Excess VRE production and export potential	13
Supplementary Note 9	Ramping requirements of hydro and thermal plants	14
Supplementary Note 10	Peak system stress	17
Supplementary Note 11	Sensitivity analysis on model resolution	19
Supplementary Note 12	Comparative cost analysis of VRE and fossil fuels	21
Supplementary Note 13	Exploiting wind resources at higher altitudes	23

Supplementary Note 1   Robustness of extreme-year synergy

The year 2024 was, by far, the driest episode in the Paute basin in more than 40 years (since 1983, the first year for which registered Paute river discharge data are available on the CELEC SUR data portal[1]), with an average discharge of 10% below that of the next-driest year (1992). This is illustrated in Supplementary Figure 1a, showing the annual average discharge across the period 1983-2024. We analysed the profile of other droughts in those years (with "dry" referring to low discharge of the river Paute, for the purposes of this explanation) by taking the 10% driest years of the full period, which (aside from 2024) were 1992, 1995, 2006, and 2010, and calculating their average seasonal discharge shortfall. We see that the shortfall across 2024 (top graph) follows a comparable cycle as in the other dry years (bottom graph), with the highest shortfall in the JAS season.
As demonstrated by ref.[2], in which hydro, solar, and wind potential data across the entire 20th century were analysed, solar and wind power potential in Latin America are largely unaffected by droughts and not linked to the El Niño-Southern Oscillation (ENSO) cycle. Climatologically, in the case of Ecuador, this can be explained as follows. With regards to solar irradiation, its spatial distribution follows east-west cloud top gradients, with modest interannual variability[3]. During El Niño conditions, changes in cloud depths that might influence incoming radiation are observed mostly on the Pacific side in western Ecuador[4], whereas the regions of highest solar power potential are located at higher altitudes in the Andes[5] (see **Supplementary Note 3**). With regards to wind, Ecuador's wind power potential is driven by ever-blowing easterlies over tropical South America (a consequence of the equatorial mid-level jet) reaching the eastern side of the Andean range[6], where they are elevated from 1000 to 3000 m.a.s.l., peaking in velocity in July and August. This results in high wind power potential at those altitudes with a robust seasonal peak (see **Supplementary Note 3**). Instrumental evidence has shown that pressure anomalies during El Niño conditions can lead to stronger-than-usual zonal pressure contrasts across the Andes and even strengthen these easterlies over Ecuador[6]. Our own analysis in **Supplementary Note 11** complements this finding: the seasonal cycle of VRE is robust across normal and dry years.
Given the above, we conclude that the extreme-year synergy identified here would generally tend to appear in extremely dry periods, and is not a coincidental occurrence from the 2024 context.

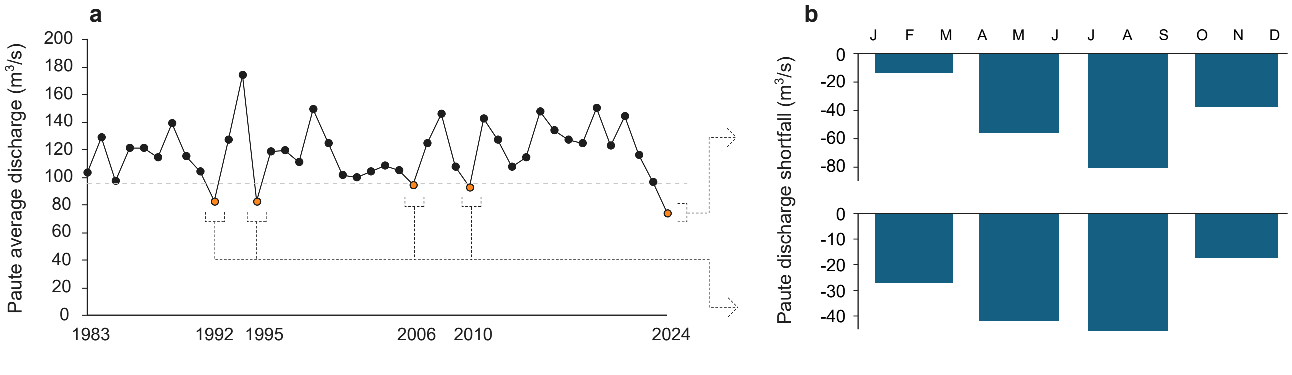

Supplementary Figure 1: **Analysing drought profiles across the last decades.** **a**, The average yearly Paute river discharge for the period 1983-2024 from ref.[1]. The dashed gray line represents the P10 value across the yearly averages. **b**, The quarterly Paute river flow shortfall for 2024 (cf. main paper, Figure 2d) compared to the average shortfall across the other drought years 1992, 1995, 2006, and 2010. The shortfall is here calculated with respect to the entire period 1983-2024 with the 10% driest years removed.

Supplementary Note 2 REVUB model calibration and validation

To calibrate REVUB for analysis of the *Complejo Paute*, we used a simple approach with only two parameters to be calibrated, one on the water regulation dimension and one on the energy generation dimension. The precise procedure was as follows. First, we calibrated the regulation fraction f_{reg} of the cascade, which is the fraction of average inflow that is allocated for storage in the reservoirs[7]. Increasing this parameter means storing more water for reservoir refill and turbining more water for reservoir drawdown; decreasing this parameter means moving closer to "run-of-river" style operation. Thus, the higher f_{reg}, the higher the amplitude in reservoir lake levels within a year. We increased the parameter f_{reg} of the cascade in increments of 0.01 until the yearly average drawdown-refill curve of lake Mazar levels (in m.a.s.l.) corresponded best to the observed values from ref.[1] in the model's calibration period, looking at the best linear fit between monthly average observed and modelled lake levels (this was found to be at $f_{reg} = 0.23$), visualised here in Supplementary Figure 2. By further increasing f_{reg}, it was observed that this was a global and not a local optimal correspondence, with the simulated drawdown-refill amplitude structurally exceeding the observed one at higher f_{reg}. The graph in Figure 3b in the paper is the corresponding full time series.

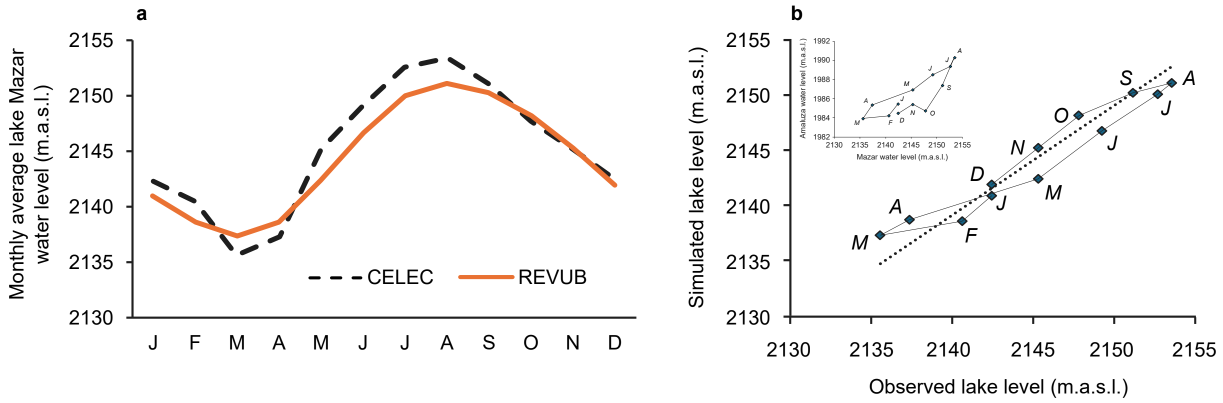

Supplementary Figure 2: **Calibration of the water regulation dimension.** **a**, The monthly average of lake level in lake Mazar across the calibration period from observations (CELEC) and model (REVUB), the latter using the optimal determined f_{reg}. **b**, The corresponding linear regression between both time series. Each dot corresponds to one month of the year (indicated by the letters), and the dotted line is the linear fit. Inset: the observed average monthly time series of levels in lake Amaluza versus levels in lake Mazar across the calibration period.

Ideally, the calibration would have been done on the basis of observed, cumulative storage volume in the cascade, but such a dataset does not exist, to our knowledge. The best alternative was to focus the calibration on lake levels of Mazar, which is by far the dominant storage in the cascade and exhibits much lower levels of "noise" in the observational time series as compared to the much smaller Amaluza. However, the calibrated $f_{reg} = 0.23$ for the cascade also gives reasonable agreement for Amaluza's average drawdown/refill cycle (see Figure 3).
The reason to use monthly averages across the model period for calibration is that this takes out the effect of incidental operational decisions in individual years, which the model would not be able to reproduce without over-fitting. The REVUB model generally assumes operation of reservoirs in a cascade to be harmonised, instead of competitive[7] (see Methods, "REVUB model implementation"). This explains the discrepancy between model and observation in certain model years, e.g. 2013, 2020 and 2022 (cf. Figure 3b of the paper). Looking at historical data[1], in those three years, discharge was relatively low in certain months at the beginning of the year compared to long-term averages (February-April in 2013; February-March in 2020; January-February in 2022). In all three cases, while levels of lake Mazar dropped, a rise in levels in lake Amaluza downstream was registered. Thus, in those cases, an operational decision was taken to empty lake Mazar for the benefit of lake Amaluza

(prioritising the filling of lake Amaluza). However, this competing operation does not appear to have been a general rule. For instance, in the first months of 2018, discharge was also relatively low, but no such decision appears to have been taken then to prioritise the refilling of lake Amaluza to the detriment of lake Mazar levels. Rather, in 2018, both lakes remained at relatively low, but more stable, water levels, and the REVUB model reproduces this harmonised operation.

Overall, modelling according to harmonised operation, as opposed to competing operation, appears justified when looking at the statistics of the average drawdown-refill cycles of Mazar and Amaluza plotted together (Supplementary Figure 2b, inset). As can be seen, both reservoirs mostly fill up and draw down simultaneously. Under drought conditions, the assumption of harmonised operation is reasonable as long as the critical volume fractions of the individual reservoirs, below which no power generation is possible, are comparable. This is the case for Mazar and Amaluza ($\sim 40\%$ for both).

The parameter f_{reg} is also directly linked to the amount of plant shutdowns occurring in the crisis year (cf. Figure 3d of the paper). This is logical, since the higher f_{reg}, the stronger the reservoir drawdown before the rainy season onset, leading to more precarious situations in years with failed rainy seasons. The above calibration procedure was seen to lead also to good agreement both in the seasonal cycle as well as in the absolute amount of plant shutdowns as a "by-product" of the lake level calibration.

In a second step, we calibrated the turbine efficiency parameter η ($0\% \leq \eta \leq 100\%$) for each hydropower plant in increments of five percentage points until the average power generation across the full model period (2011-2024) agreed best with the observed data[1]. In this case, for instance, at $\eta = 80\%$, agreement between REVUB and observed data was within 1.8% for Paute-Molino (an average of 5296 GWh/year according to REVUB, versus 5203 GWh observed). Note that this step only calibrates the absolute level of power generation and has no influence on the seasonal shape nor the interannual variability of power generation. Note, further, that for run-of-river plants—such as Sopladora in the case of the *Complejo Paute*—the parameter f_{reg} does not apply, and hence η was the only calibration parameter for such plants (but the f_{reg} of the upstream reservoirs, of course, does influence the power generation profile at Sopladora).

Multiannual time series on lake levels—for the simulated plants with reservoirs, i.e. Mazar (lake Mazar), Paute-Molino (lake Amaluza), and Marcel Laniado (lake Daule Peripa)—are given in Supplementary Figure 3. We note that agreement between simulations and observations is overall encouraging, especially on the average seasonal drawdown-refill-curves. The largest discrepancies are found for Amaluza, the smallest of the three reservoirs (both in absolute terms, as well as compared to river discharge) with a relatively high amount of "noise" in the observational time series. We find the discrepancies are mainly due to (i) uncertainties around the coordination between Mazar and Amaluza (see the discussion above) and (ii) uncertainties in literature regarding the precise area-volume-elevation curve of lake Amaluza, which according to ref.[8] has been subject to substantial change over time due to sedimentation affecting the relatively small reservoir.

In Supplementary Figure 4, we show the match between simulations and observations of annual power generation from the hydropower plants not shown in Figure 3 of the paper, including plants outside of the *Complejo Paute*. In general, the match between observations and simulations is encouraging. It is worth highlighting the Coca Codo Sinclair plant (Supplementary Figure 4d), Ecuador's largest in terms of capacity (cf. Figure 1 of the paper). Its production profile (Supplementary Figure 4d, inset) is almost baseload-like. This is a design feature: a discharge of 222 m^3/s is diverted from the Coca river into a tunnel to an underground powerhouse several hundred meters lower, from where the captured and turbined discharge is returned to the river[10]. Since the Coca river's discharge is usually above this design discharge of 222 m^3/s, the production profile of the plant is quite unseasonal, with usually only a dip in November, when river discharge tends to fall below this design flow. Another example to highlight is Delsitanisagua, which shows the largest discrepancy between simulation and observation (Supplementary Figure 4f), specifically in the first year of operation (2019). Analysing the seasonal profile (Supplementary Figure 4f, inset) reveals that some turbines were inactive for part of that year, and for the months of August and September, the plant was fully idle. This was due to maintenance and finalising civil works[11], explaining the discrepancy.

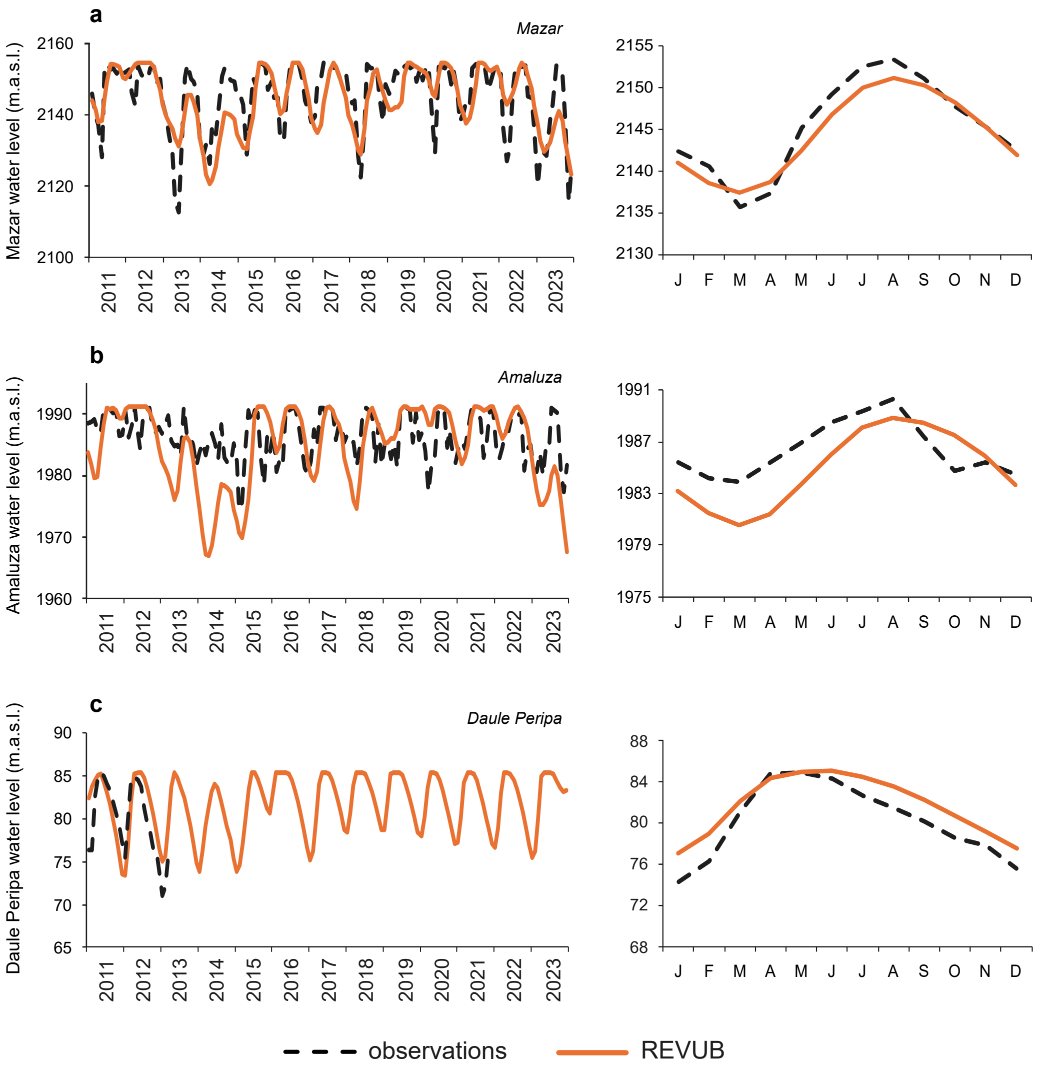

Supplementary Figure 3: **Reservoir level curves from simulated hydropower plants compared to observations**. The chart shows the time series (left) and average seasonal curves (right) across the calibration period of lake levels for **a** lake Mazar, **b** lake Amaluza, and **c** Daule Peripa reservoir (of the Marcel Laniado hydropower plant) under scenario *S1 regular*. Lake levels observations are from CELEC (ref.[1] for **a-b**, and ref.[9], only available until March 2013, for **c**). *m.a.s.l.* = metres above sea level.

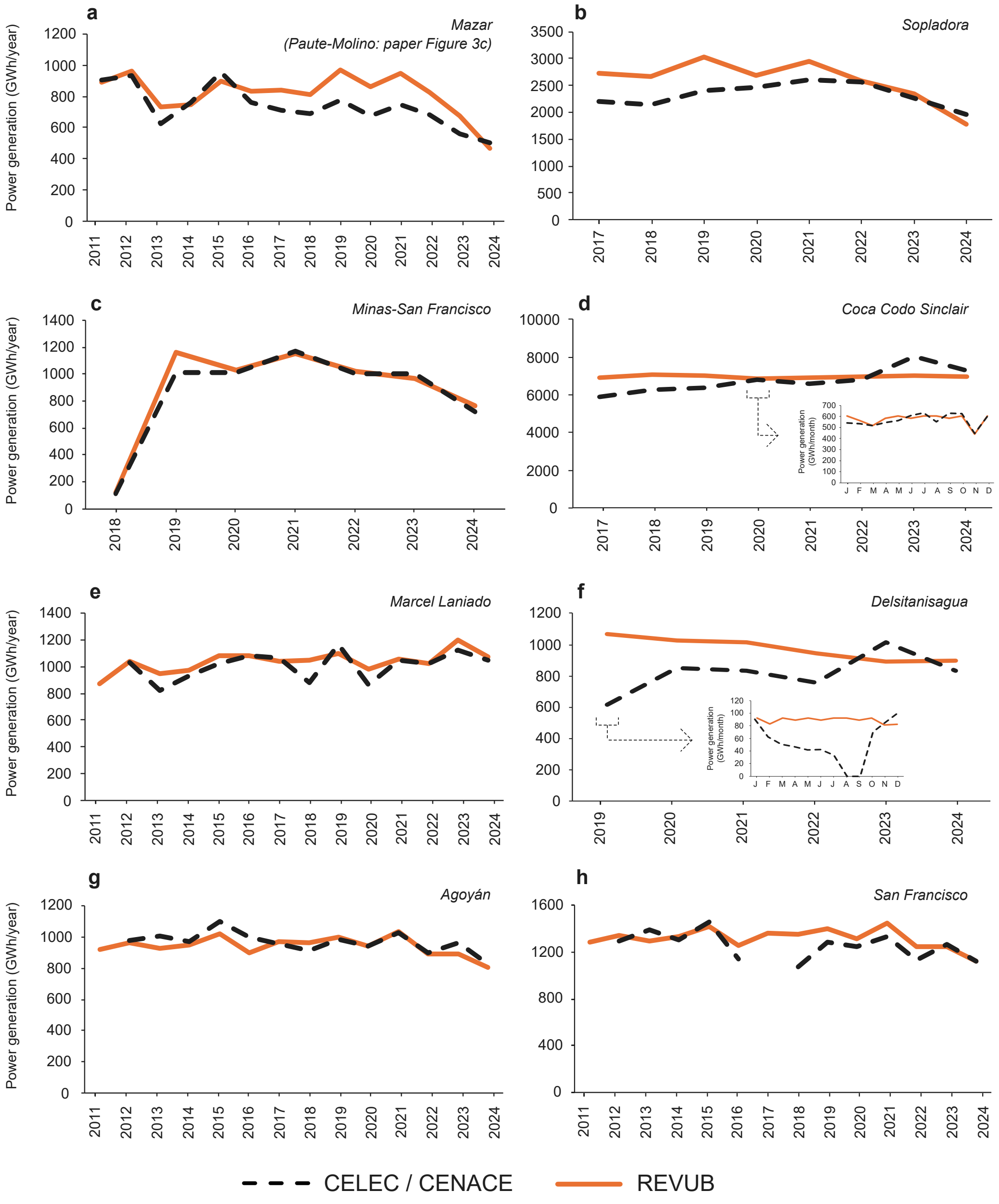

Supplementary Figure 4: **Power generation curves from simulated hydropower plants compared to observations.** Yearly power generation according to REVUB simulations and observations for **a** Mazar (*S1 regular*), **b** Sopladora (*S1 regular*), **c** Minas-San Francisco, **d** Coca Codo Sinclair, **e** Marcel Laniado (simulation according to *S1 regular*), **f** Delsitanisagua, **g** Agoyán, and **h** San Francisco. (Note: for the pure run-of-river plants, the scenario choice is irrelevant as those plants do not have the flexibility to be hybridised with hydropower.) Observations from CELEC[1] for **a-c** and from CENACE[12] for **d-h**. Note that, in some cases, one or two years are missing from the observational time series. Inset to **d**: Comparison of seasonal profile between simulations and observations for Coca Codo Sinclair for 2020. Inset to **f**: Comparison of seasonal profile between simulations and observations for Delsitanisagua for 2019.

Supplementary Note 3 Geospatial representation of VRE

The geospatial distribution of the most attractive locations in Ecuador for VRE plants according to the Model Supply Regions methodology[13,14] as implemented here (see Methods, "Solar and wind power data" in the main paper) is given in Supplementary Figure 5. The circle of 200 km radius centered around the *Complejo Paute* shows which clusters of VRE resources were included in the analysis.
The 200 km radius was chosen for practical considerations: the *Complejo Paute* is managed by the CELEC SUR business unit, which also contains the Minas-San Francisco hydropower plant on the other side of the Andean divide[1]. The distance between the *Complejo Paute* and the Minas-San Francisco plant is roughly 200 km; for hydro-VRE hybridisation to be effectively integrated into daily reservoir operation, we consider that the VRE plants should ideally be housed under the same business unit as the hydropower plants. Thus, we consider a 200-km distance as a benchmark for finding solar and wind power sites to integrate with the *Complejo Paute* as realistic. This seems reasonable also given the locations of Ecuador's existing wind farms, also shown in Supplementary Figure 5.
For wind power, a distinction is made between resources in locations below 2000 m.a.s.l. (the primary sub-selection) and an extended set of locations below 3000 m.a.s.l. For both solar and wind power, the most attractive locations are seen to be found across the Andean mountain range traversing the country longitudinally, where solar irradiation and wind speeds are highest and the transmission grid is never too distant.
The inset graph shows the seasonal profile across the primary and extended sub-selections of wind

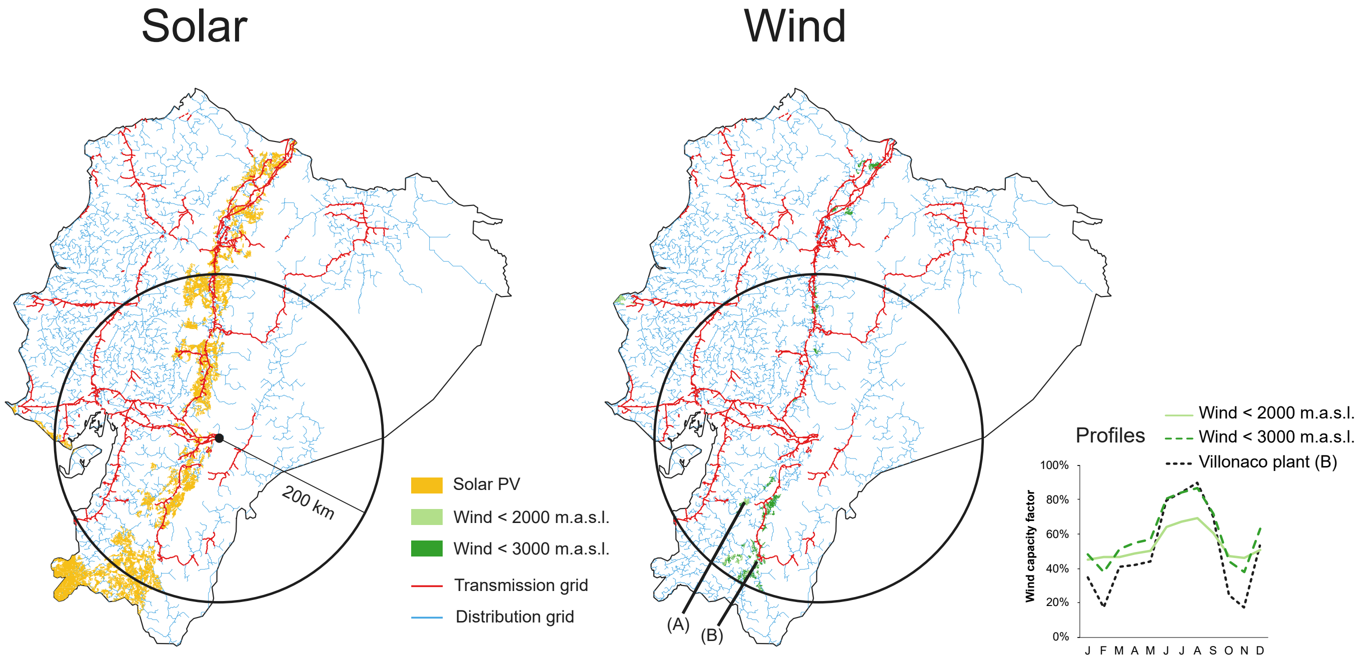

Supplementary Figure 5: **Ecuador's most attractive solar and wind power locations.** The map shows the study area and the geographic location of Ecuador's solar and wind Model Supply Regions[13,14] for both solar and wind power. The selection of candidates for hydro-VRE hybridisation is limited by the circle centered on the *Complejo Paute*. Transmission and distribution grid lines are taken from GridFinder[15,16], and the country shapefile from ref.[17]. On the wind map, the locations are indicated of (A) the 50-MW Minas de Huascachaca wind power plant and (B) the 16.5 MW Villonaco wind power plant. Inset: Seasonal average wind power profiles across all locations below 2000 and 3000 metres above sea level (*m.a.s.l.*) for the meteorological year 2018. The observed Villonaco plant curve is taken from ref.[18] and also represents the year 2018.

power locations. Clearly, while higher elevations tend to result in somewhat higher average capacity factors, especially in the strongest wind season, it also leads to seasonal variability with a higher amplitude. Due to this trade-off, it is ambiguous whether the inclusion of such locations leads to more promising hydro-VRE hybridisation outcomes; cf. **Supplementary Note 13** for an analysis.
For comparison and validation, we also show the monthly capacity factor curve published for the 16.5 MW Villonaco wind power plant[18] (located at ~2650 m.a.s.l.) for the meteorological year 2018. This curve follows the modelled profile of the sub-selection of MSRs quite accurately, especially during the highest-yield months. (Note that the Villonaco site has wind turbines with 65-m hub height, whereas the MSRs are modelled for 100-m hub heights.)

Supplementary Note 4 Solar-driven "micro-recoveries"

We coin the term "micro-recoveries" in the paper to illustrate that the differences in storage filling levels between morning and mid-day in a scenario with hybridised hydro-solar operation (S2) are small, but nevertheless consequential. In our simulations, the differences in lake filling levels between morning and mid-day in S2 lie in the order of 0.1% (Figure 4 of the paper). While this may not seem much, it is enough to buy the reservoirs several additional hours to generate power each day (albeit not at full capacity, with only a limited number of turbines running) before water levels are back at where they were. A back-of-the-envelope calculation can illustrate this:

- If the daily, temporary boost of the storage filling level facilitated by solar power under scenario S2 is roughly 0.1%, this corresponds to a total water volume of 0.1% $\times$ 530 Mm^3 = 530.000 m^3 (with 530 Mm^3 the cumulative storage capacity in the *Complejo Paute*).
- The amount of water allocated to the regulation function of the reservoirs (i.e. the water stored and later released) is in the order of $f_{reg} \cdot Q_{av} \approx 29$ m^3/s, with $f_{reg} = 0.23$ the regulation fraction of the cascade (see **Supplementary Note 2**) and $Q_{av} \approx 123$ m^3/s the long-term average river discharge[1].
- Since 530.000 m^3 / (29 m^3/s) $\approx$ 5.2 hours, the additional water volume saved during the solar ramp-up could cover around five hours of average regulation outflow once solar starts ramping down. Under prudent operation, at somewhat lower-than-average regulated outflow, this amount of hours could be extended.
- While this may not be sufficient to cover the full daily cycle until solar PV ramps up again, it is still a considerable improvement compared to a situation without hydro-solar hybridisation. The extra hours of power generation translate directly into a lower overall duration of forced plant shutdowns.

The crucial element generating the operational benefits here is the fact that the solar power generation cycle repeats every day. Thus, the amount of water saved inbetween two consecutive solar production cycles need not necessarily be large in order to produce these benefits—merely sufficient to cover periods in the order of hours.

Supplementary Note 5 Solar-wind synergies

The combination of solar and wind power in the proposed VRE portfolios allows exploiting synergies between the two resources that are beneficial to hydro-VRE hybridisation. In Supplementary Figure 6, we show the average and interquartile range of capacity factor curves at hourly level for each month of the year according to the MSR analysis for the sub-selection of solar and wind power sites shown in Supplementary Figure 5.

It can be seen that solar-wind synergies fall mainly into two categories, depending on the season: (i) wind peaking several hours after the solar peak at mid-day (roughly October to April); (ii) wind peaking a few hours before the solar onset in the morning (roughly May to September). The stability factor C_{stab} proposed by ref.[19], measuring the quality of diurnal solar-wind synergies, is higher than 0.5 for nearly all months of the year (assuming an equal installed capacity for solar and wind power, as in this study), signifying high complementarity between the resources[20].

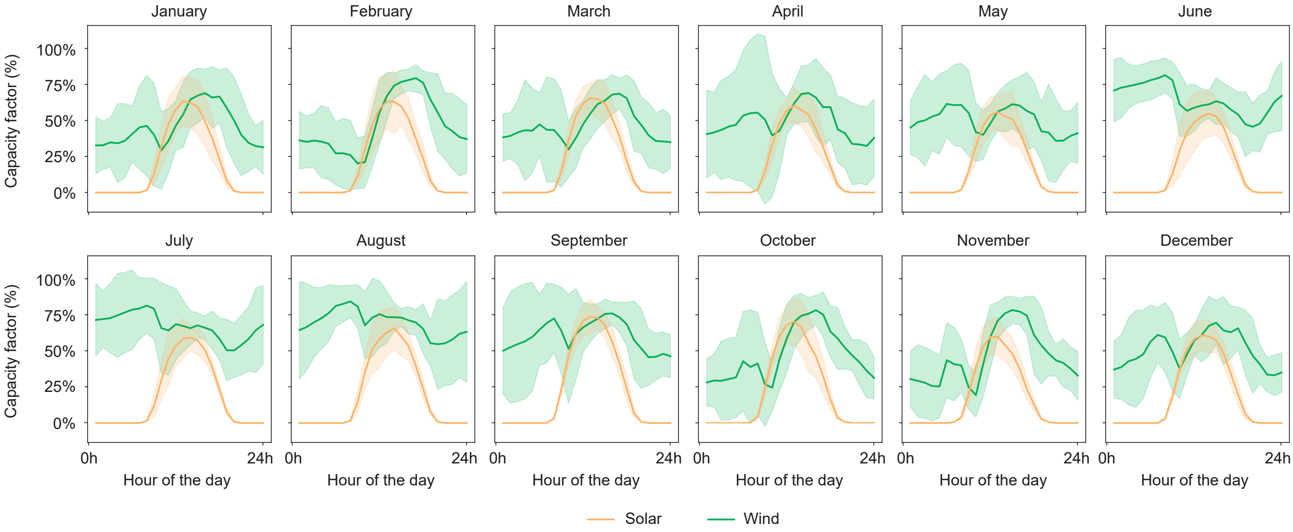

Supplementary Figure 6: **Illustration of synergies between solar and wind power in Ecuador.** The capacity factor average (lines) and interquartile ranges (shaded areas) for each hour of the month across the sub-selection of locations shown in Supplementary Figure 5, based on the meteorological year 2018. For wind, the graphs refer to the sub-selection of locations below 2000 m.a.s.l.

Supplementary Note 6 Regular versus prudent reservoir operation

Moving to more prudent overall hydropower reservoir management—deliberately keeping more water in the reservoir in general, thereby foregoing part of the hydropower generation as well as of the buffer capacity for the yearly refilling cycle—can lead to better results in terms of overcoming unforeseen dry periods. In Supplementary Figure 7, we show the time series of water storage levels in the *Complejo Paute* under regular scenario S1 (identical to the results for S1 in Figure 4a of the main paper) alongside the same data for a prudent variation of that scenario. As opposed to the regular operation, the prudent variant keeps water levels manageably high throughout 2023, allowing to start the second successive dry year 2024 in much better conditions. Although the simulation is myopic throughout 2024, the prudent variation ensures substantially higher lake levels compared to regular operation in the crisis year, thus avoiding emergency plant shutdowns in the *Complejo Paute*.
As the reservoirs are generally fuller under prudent operation, major peaks in reservoir outflow may—depending on whether the year in question is hydrologically dry or wet, and on what the precise threshold for "peak" is—occur somewhat more often (0%-∼20%) under prudent operation as compared to regular operation. However, that does not make these events more *intense* as measured in m^3/s. In individual months, prudent operation sometimes shows somewhat higher maximum discharge values than regular operation, but on annual average basis, we observe that the maximum downstream discharge values are practically unchanged between regular and prudent scenarios. These values occur in the moments when the river carries maximum discharge into the reservoir while the latter is nearly full, and those single moments are practically identical between regular and prudent operation (cf. the months with highest filling levels in Supplementary Figure 7).

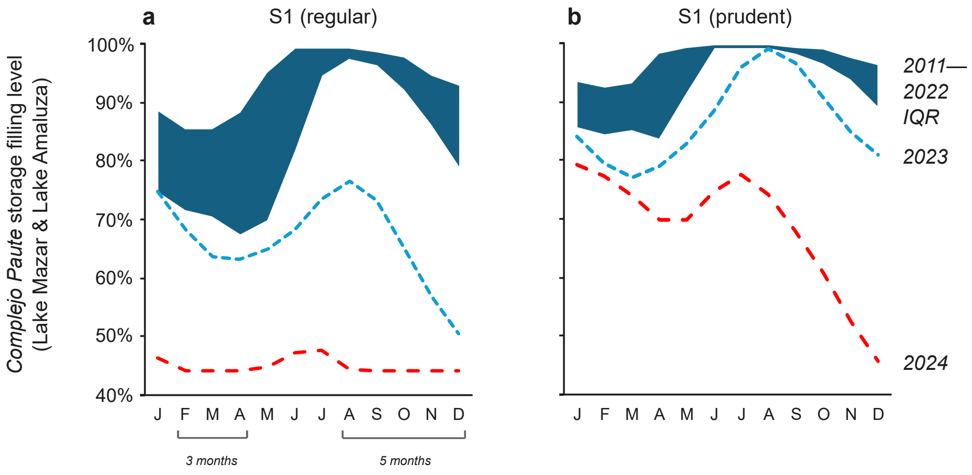

Supplementary Figure 7: **Regular and prudent reservoir operation.** Simulation outcomes showing time series of the water storage level in the *Complejo Paute* under regular and prudent variants of scenario S1. Shown are the interquartile range (IQR) of monthly averages for the period 2011-2022, as well as the monthly averages for 2023 (fine blue dashes) and 2024 (rough red dashes). The critical months in 2024, defined as having median storage levels (calculated across all hours in a month) within 0.10 percentage points of the minimum operational level (which lies just below 42%), are shown on the horizontal axes declining from a total length of 8 months (regular) to zero (prudent).

Supplementary Note 7 Stop-restart hydropower cycles during the crisis peak

In Figure 5b-c of the main paper, REVUB simulation outcomes include the onset of stop-restart cycles of hydropower generation in the *Complejo Paute* in the latter part of 2024. These cycles did in fact occur during the crisis: the hydropower plants stopped production for periods of one to several hours before restarting, as shown in Supplementary Figure 8.

These cycles are modelled in a purely physical manner in REVUB[7]: the model forces hydropower generation to stop when critical lake levels are reached, and restarts generation once these levels recover. The mentioned critical levels are defined separately for each hydropower plant, through a model input parameter which represents the minimum filling level (fraction of reservoir volume) of each hydropower reservoir, below which turbine operation is no longer possible. The critical values used for the Mazar and Amaluza reservoirs in our study are close to 40% for both, and are derived from maximum and minimum operational water levels converted to volumes using the reservoir bathymetry curves (for full references, see the REVUB input dataset given on Zenodo[21]).

In reality, the stopping and restarting of reservoir releases in critically dry periods is more complex and dynamic in character than a simple boolean based on storage levels, with e.g. decisions being made on the basis of the societally least damaging timing of load shedding[1]. Nevertheless, it is encouraging to see that the modelling framework with this simplified representation can not only reproduce yearly and monthly production and shutdown statistics (cf. main paper, Figure 3), but is also capable of reproducing, at least conceptually, this stop-restart element at hourly scale.

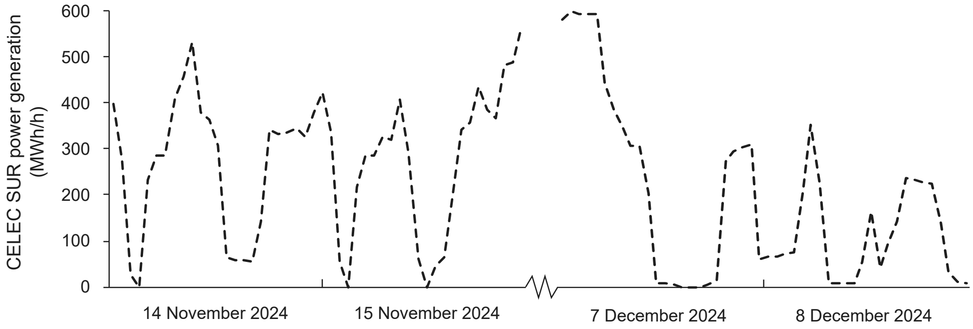

Supplementary Figure 8: **Stop-restart cycles of hydropower operation in the last months of 2024.** Shown are operational data from CELEC[1] of hydropower generation at Mazar, Paute-Molino, Sopladora, and Minas-San Francisco plants (together forming the CELEC SUR portfolio) for two example periods of two consecutive days in latter 2024.

Supplementary Note 8 Excess VRE production and export potential

In Supplementary Figure 9, we show a visualisation of the maximum hourly (Supplementary Figure 9a) and aggregated monthly (Supplementary Figure 9b) surpluses for scenarios S1 and S4 under their regular and prudent variants, assuming hydropower generation follows either the hydrologically normal year 2022 or the crisis year 2024. (Visualisations of the corresponding seasonal and hourly power mix are given in Figure 5b-c of the paper for *S1 regular* and *S4 prudent* under 2024 hydrology.) The excess production appears mainly in the months of maximum hydropower generation. In this example, the maximum instantaneous surplus (Supplementary Figure 9a) would be around 920 MW (400 MW) in a normal (dry) year under scenario *S1 regular*, rising to around 1420 MW (780 MW) under scenario *S4 prudent*, implying that roughly 500 MW (380 MW) of this surplus can be attributed to VRE integration. The aggregated excess generation (Supplementary Figure 9b) would correspond to around 20% (4%) of annual VRE production in a normal (dry) year, concentrated in certain months.
However, these excess rates only hold when assuming that demand will not further rise in the future, which—given historical data—appears highly unlikely. Power generation in Ecuador has, across the last 25 years, had to increase at an average rate of 6% per year to keep up with demand[12]. Any further rise in overall demand would directly lead to lower excess rates for VRE, and it is thus likely that, in the near future, the proposed quantities of VRE would be able to be near-fully absorbed by Ecuadorian electricity demand. Even if this demand increase were to not materialise, the additional capacity and generation excess caused by VRE still falls well within what e.g. the planned new 1000-MW interconnector to Peru could export. Moreover, in Peru, the low season of hydropower (which represents more than half of Peru's electricity mix) coincides well with the periods of hydro-VRE excess identified here for Ecuador[22]. Hence, there is reason to think that these excesses could hold export opportunities for Ecuador. Even under the worst possible outcome, which would be curtailment of all the excess VRE, the cost outlook for VRE as compared to new fossil-based electricity would still be positive, as is discussed in more detail in **Supplementary Note 12**.

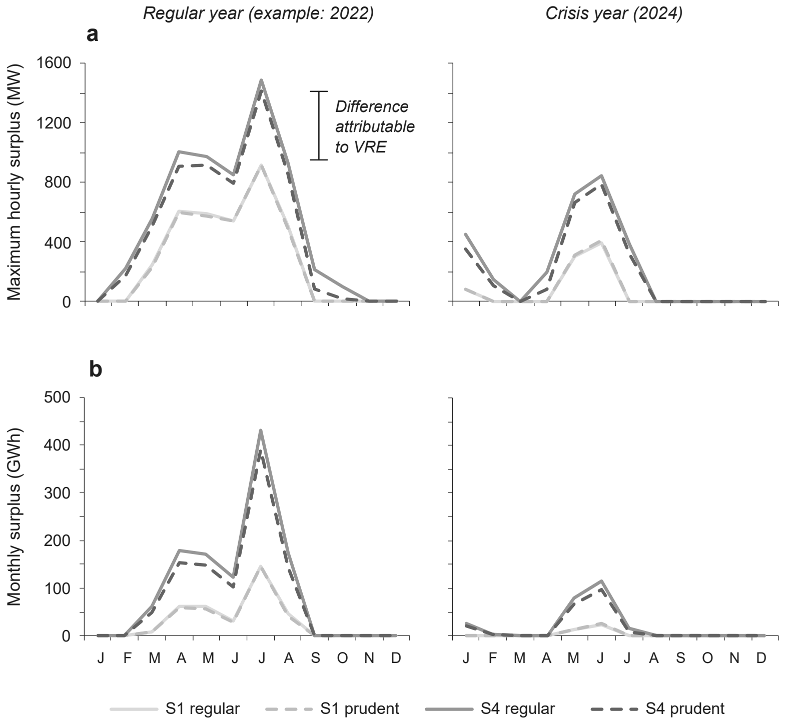

Supplementary Figure 9: **Estimation of excess production compared to demand. a**, The maximum instantaneous hourly surplus, for a hydrologically normal year (2022, left) and the crisis year (2024, right) for S1 and S4. **b**, Idem for the accumulated monthly surplus.

Supplementary Note 9 Ramping requirements of hydro and thermal plants

This section presents an analysis of the hourly ramping requirements for both hydropower and thermal/imports under different scenarios. In Supplementary Figure 10, we show the average hourly ramping envelopes (in MW) for hydropower and for thermal & imports, for the hydrologically normal year 2022 and for the crisis year 2024, comparing two scenarios (*S1 regular* and *S4 prudent*; cf. Figure 5b-c of the paper). We observe, as expected, that the average ramping requirements for hydropower increase with VRE integration (compare Supplementary Figure 10a, *S1 regular* versus *S4 prudent* for 2022), but also that these ramping requirements are of the same order as those caused by the hydropower plant shutdowns in the crisis year (in Supplementary Figure 10a, the curve for *S1 regular* for 2024, with hydro shutdowns but without VRE, has comparable average ramping across one hour as *S4 prudent*, with mitigated hydro shutdowns but with high VRE contributions). Concerning thermal power and imports, the ramping envelope is not affected much by the integration of VRE (compare Supplementary Figure 10b, *S1 regular* versus *S4 prudent* for 2022), which is logical as the VRE variability is mostly taken care of by hydropower in our scenarios. For the crisis year, their ramping requirements are seen to increase slightly for *S1 regular*, as thermal power plants have to perform extra ramping to deal with the frequent hydropower plant shutdowns, but the effect is relatively small (compare Supplementary Figure 10b, *S1 regular* for 2024 versus 2022).
Such curves allow to calculate ramping needs as expressed in percentage of available capacity per hour. We present the results in Supplementary Figure 11, for average (Supplementary Figure 11a) and maximum (Supplementary Figure 11b) ramping needs. We observe that the average ramping needs for both thermal and hydropower are in realistic ranges, below ∼10% of their respective total capacity per

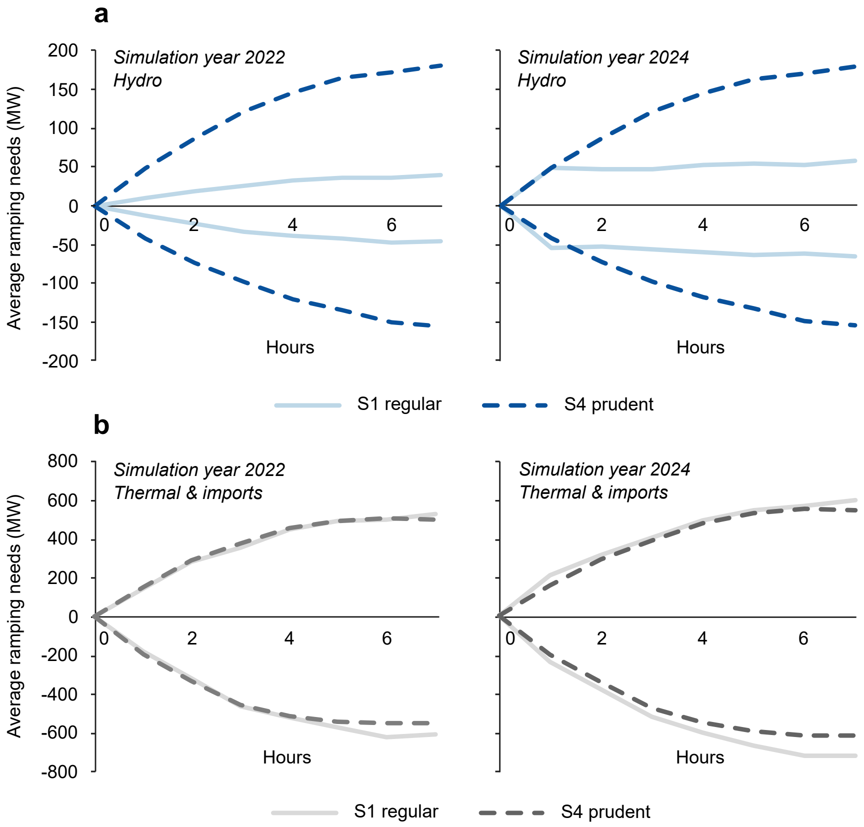

Supplementary Figure 10: **Ramping envelopes for hydropower and thermal/imports.** **a**, Average ramping envelopes for hydropower for the regular year 2022 and the crisis year 2024, for *S1 regular* and *S4 prudent*. Plotted are the average positive and negative ramping requirements across time spans of one to seven hours. **b**, Idem for thermal power and imports.

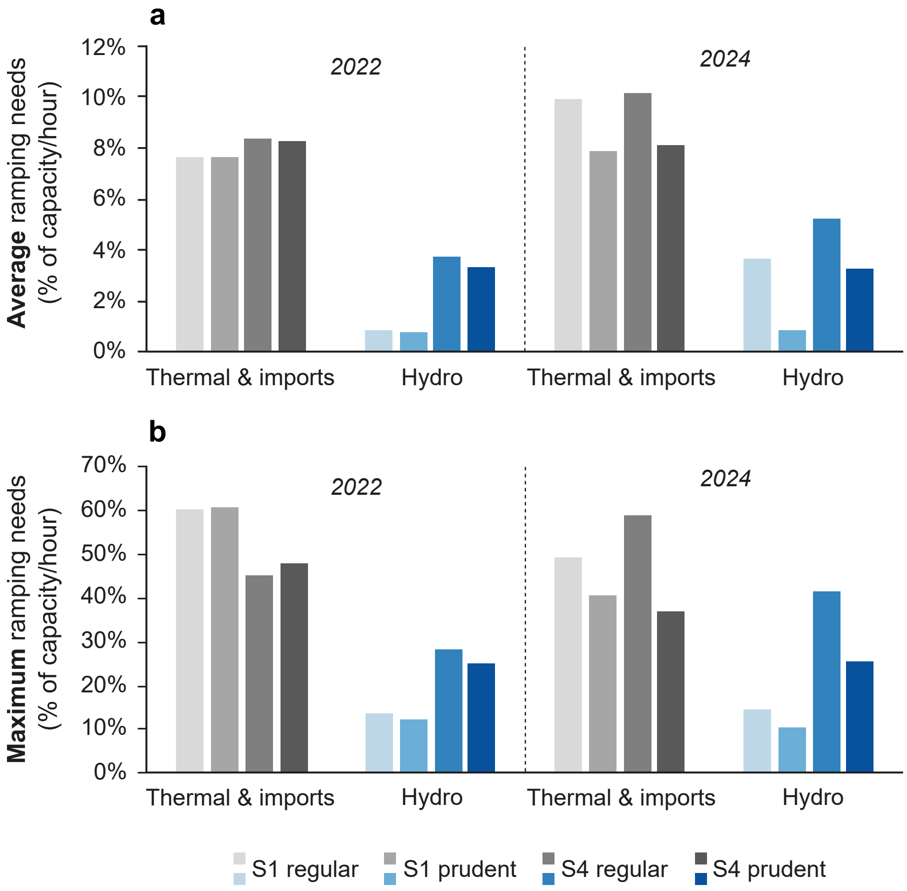

Supplementary Figure 11: **Average and maximum hourly ramping requirements.** **a**, Average ramping requirements for hydropower and thermal power & imports for the normal year 2022 and the crisis year 2024 under the regular and prudent variants of S1 and S4. The requirements are expressed in %/hour of total capacity with ramping capabilities. For hydropower, in our simulations, this is the sum of the installed capacity of Mazar (170 MW), Paute-Molino (1100 MW), and Marcel Laniado (213 MW). For thermal power & imports, this is the sum of existing thermal capacity (1862 MW) and interconnection capacity (450 MW with Colombia and 80 MW with Peru). **b**, Idem, but for *maximum* hourly ramping requirements.

hour (Supplementary Figure 11a). Further, prudent operation lowers these needs somewhat, especially in crisis years as hydropower plant shutdowns are avoided (compare Supplementary Figure 11a, *regular* versus *prudent*). Insofar as maximum ramping needs are concerned (Supplementary Figure 11b), these are logically substantially higher independently of scenario and year, but it concerns events that only happen a few times a year, and the needs remain within available capacity limits. Given typical maximum ramp rates of thermal and hydropower plants, which can reach more than 10% of capacity per minute[23], these per-hour values seem reasonable. Interestingly, while the introduction of VRE increases the average ramping requirements for thermal and imports slightly (compare Supplementary Figure 11a, S1 versus S4), it *lowers* the *maximum* ramp rates of thermal power in regular years (compare Supplementary Figure 11b, S1 versus S4 for 2022). This reflects once more the idea that VRE obtains a substantial "capacity credit" through being hydro-backed.

We note that the amount of startups and shutdowns of thermal power and imports are quite comparable under the investigated scenarios, as shown in Supplementary Figure 12 for 2022 and 2024. Under normal (2022) conditions, the number of startups/shutdowns is logically higher than under crisis (2024) conditions, as there are more moments of the year in which hydropower can fully cover demand. While VRE integration increases the amount of startups/shutdowns somewhat, the increase

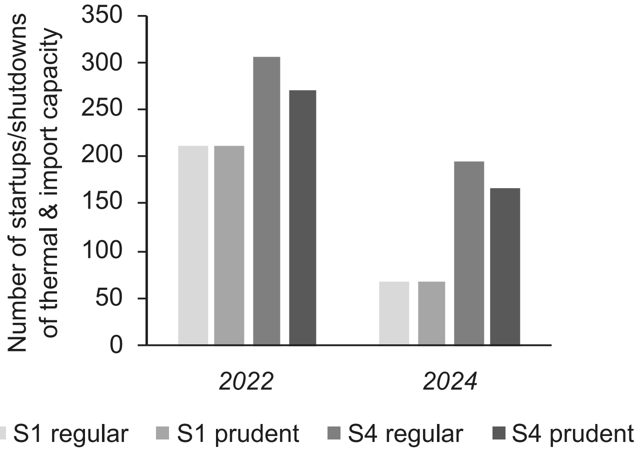

Supplementary Figure 12: **Start-ups and shutdowns of thermal/import capacity.** The chart shows the implied amount of start-ups and shutdowns of thermal and import capacity needs for the hydrologically normal year 2022 and the crisis year 2024 in our simulations, under the regular and prudent variants of scenarios S1 and S4.

between *S1 regular* and *S4 prudent* for the normal year 2022 is only 25%. Costs of increased cycling—manifesting in more startups and shutdowns as well as more wear and tear to equipment—are reported by IRENA[23] to lie in the order of 1 USD/MWh for combined-cycle gas power plants. This is less than 1.5% of the fuel costs of natural gas paid by CELEC[24], and increasing these costs by 25% would increase the expected levelised cost of electricity (LCOE) for gas power plants in Ecuador by less than 1% (see **Supplementary Note 12**).

Supplementary Note 10 Peak system stress

In Supplementary Table 1 below, we present the power mix composition of Ecuador in the single hours of peak system stress (in terms of the capacity deficit) during the simulated year 2024, as well as in the hours with second-high system stress. The numbers illustrate that there are three contributions to reducing the capacity deficit, going from scenario *S1 regular* to scenario *S4 prudent* (cf. Figure 6b of the paper):

- The direct contribution of VRE in the hours of peak stress, in this case wind power since the moments of peak stress occur after sunset (compare *S1 regular* to *S4 regular*). However, this contribution is "coincidental" and not necessarily guaranteed in the moments of highest stress.
- The contribution of increased hydropower availability due to prevented plant shutdowns, especially of the powerful Paute-Molino plant (compare *S1 regular* and/or *S4 regular* to *S1 prudent*). However, it must be noted that preventing a shutdown does not mean that the plant is available at full capacity; rather, in the case of Paute-Molino, it would run one or two turbines out of the ten installed.
- The indirect contribution of hydro-VRE synergies having led to increased water storage throughout the weak rainy season (cf. Figure 4a of the paper), allowing to assure higher hydropower availability for the moments of highest system stress later in the year (compare *S1 prudent* to *S4 prudent*). For this effect, it is not the VRE availability *in the moment* that leads to the reduced capacity deficit, but its synergies with hydropower *earlier in the year.*

In the paper, the concept of the "virtual" or "indirect" capacity credit of hydro-backed VRE is considered to reflect all three of the above effects within a single indicator, noting that this capacity credit requires appropriate (prudent) hydropower operation to manifest as a precondition, which is then further enhanced through hydro-VRE synergies.
A graphical representation of the hourly power generation mix during the days of highest and second-highest capacity deficit indicated in Supplementary Table 1 is provided in Supplementary Figure 13 for all four presented scenario variants.

**Scenario**	**Moment of system stress** day & hour	**Stress rank**	**Hydro-power** MW	**VRE** MW	**Thermal & imports** MW	***Capacity deficit*** *MW*	**Demand** MW
S1 regular	26/11/2024 20:00	1	1252	0	2392	*1210*	4853
S1 prudent	05/11/2024 20:00	1	1468	0	2392	*999*	4860
S4 regular	05/11/2024 20:00	1	1215	167	2392	*1086*	4860
S4 prudent	26/11/2024 20:00	1	1668	214	2392	*580*	4853
S1 regular	18/11/2024 20:00	2	1213	0	2392	*1157*	4761
S1 prudent	26/11/2024 20:00	2	1467	0	2392	*994*	4853
S4 regular	26/11/2024 20:00	2	1215	244	2392	*1003*	4853
S4 prudent	19/11/2024 19:00	2	1658	218	2392	*539*	4807

Supplementary Table 1: **Power mix at peak system stress.** The table shows the composition of the power mix in moments of peak system stress under the scenario variants shown in Figure 6a of the paper, and the corresponding capacity deficits. "Stress rank" indicates whether it concerns the moment of highest system stress of the year within each scenario variant (1), or the moment with second-highest system stress (2). The capacity deficits in the worst moments (stress rank 1) are visualised in Figure 6b of the paper.

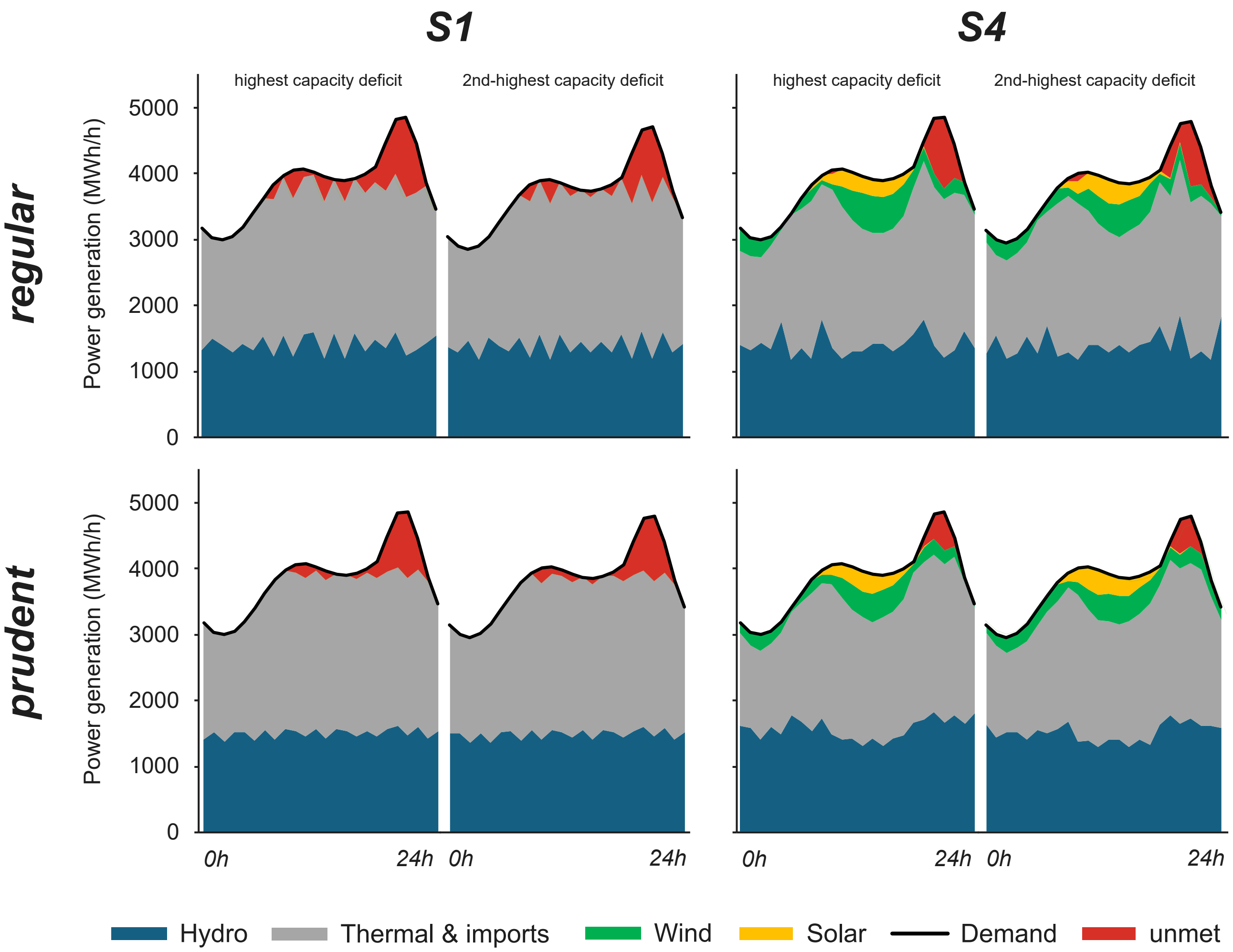

Supplementary Figure 13: **Hourly power generation on the days of highest capacity deficit.** The chart shows the hourly generation mix on the days containing the hours of highest system stress (measured by the capacity deficit) for the four scenario variants presented in Supplementary Table 1, as well as on the days containing the hours of second-highest system stress.

Supplementary Note 11 Sensitivity analysis on model resolution

We ran sensitivity studies related to the resolution of REVUB model input data to show the difference between (i) the use of monthly averaged versus daily-resolution river flow, and (ii) the use of a single weather year (2018) at hourly resolution versus the full modelling period.

On (i), we note that in terms of sub-daily operation, the reservoirs in the *Complejo Paute* are large enough to buffer typical differences appearing between monthly- and daily-resolution datasets. The combined storage capacity in the cascade is 50 days (39 days from Mazar, plus 11 from Amaluza) of average river discharge, i.e. more than 1.5 months of average river flow. Thus, one would expect that the choice of river flow resolution does not affect the principal conclusions of the study.

On (ii), it is important to understand that interannual variability and spread in seasonal variability of VRE is vastly inferior to that of river flow (Supplementary Figure 14), and that there is no discernible correlation between hydrological conditions and solar/wind resource quality across different years (Supplementary Figure 15), including in very dry years.

The latter is consistent with ref.[2], who concluded, on the basis of analysing hydro, solar and wind potential data across the entire 20th century, that solar and wind power potential across Latin America is largely unaffected by the El Niño-Southern Oscillation (ENSO) cycle (see **Supplementary Note 1)**. Based on this information, one would expect that the weather year choice also does not affect the principal conclusions of the study.

Below, we show in tabular format (Supplementary Table 2) the most important output parameters for three sensitivity studies: (i), (ii), and (i) + (ii). We note that the main conclusions of the paper indeed remain unchanged.

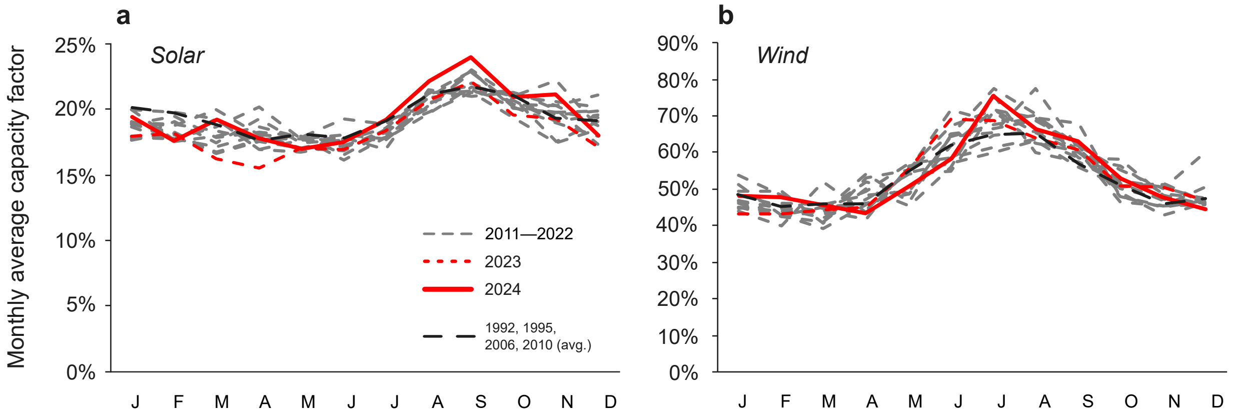

Supplementary Figure 14: **Interannual and seasonal variability in VRE generation. a**, The average monthly capacity factor of solar power plants across the analysed locations, for all years in the modelling period. The dry year 2023 (red, dashed) and the extremely dry year 2024 (red, thick) are highlighted to allow comparison to Paute and Jubones river discharge in Figure 2a-b of the paper. An average curve across the other very dry years (1992, 1995, 2006 and 2010) identified in Supplementary Figure 1 (outside the modelling period) is shown in dashed black. **b**, Idem for wind power. Data obtained using Model Supply Regions methodology[13,14] for the locations described in **Supplementary Note 3**.

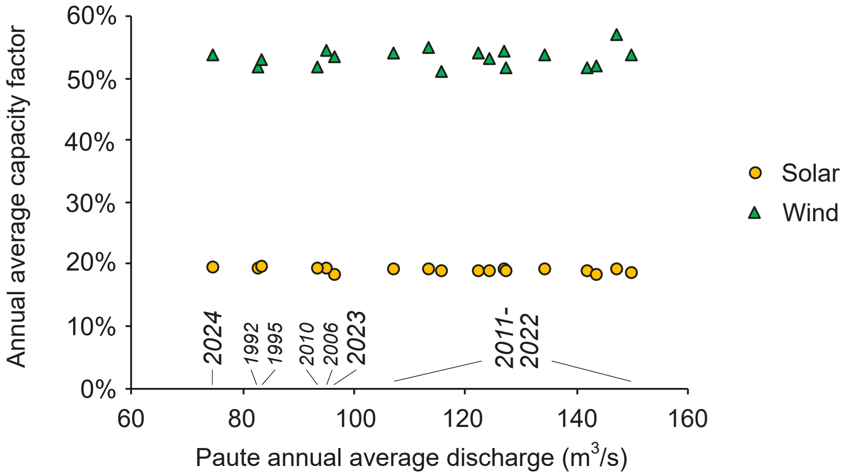

Supplementary Figure 15: **Lack of correlation between hydropower and VRE conditions.** The average yearly capacity factor of solar and wind across the studied locations plotted against the average yearly Paute river discharge[1] for the period 2011-24, as well as for the other very dry years (1992, 1995, 2006 and 2010) identified in Supplementary Figure 1.

**Parameter**<br>*River flow resolution (i)* → *VRE years (ii)* →	**Main study**<br>*Monthly*<br>*One year*	**Sensitivity**<br>*Daily*<br>*One year*	**Sensitivity**<br>*Monthly*<br>*Full period*	**Sensitivity**<br>*Daily*<br>*Full period*	**Actual value**
**Results on *Complejo Paute***					
Paute-Molino avg. production 2011-23 (*S1 reg.*)	5452 GWh	5087 GWh	5452 GWh	5087 GWh	5308 GWh[1]
Paute-Molino production 2024 (*S1 reg.*)	3268 GWh	3211 GWh	3268 GWh	3211 GWh	3843 GWh[1]
Paute-Molino shutdown duration 2024 (*S1 reg.*)	56.6 days	62.6 days	56.6 days	62.6 days	57.3 days[1]
Paute-Molino shutdown duration 2024 (*S4 reg.*)	30.0 days	31.6 days	31.3 days	30.2 days	-
Complejo Paute hybridisable VRE (*S4 reg.*)	1226 MW	1165 MW	1179 MW	1115 MW	-
Complejo Paute hybridisable VRE (*S4 prud.*)	1075 MW	1022 MW	1047 MW	993 MW	-
**Results on Ecuador power mix**					
Capacity deficit in 2024 (*S1 reg.*)	1210 MW	1140 MW	1210 MW	1140 MW	1298 MW[25]
LOLE in 2024 (*S1 reg.*)	7.4%	7.3%	7.4%	7.3%	-
LOLE in 2024 (*S4 prud.*)	1.2%	1.4%	1.3%	1.5%	-
Reduction capacity gap (*S1 reg.* → *S4 prud.*)	−52%	−47%	−56%	−50%	-
Reduction generation gap (*S1 reg.* → *S4 prud.*)	−90%	−87%	−90%	−87%	-
Capacity credit of VRE (*S4 prud.*)	59%	52%	65%	58%	-

Supplementary Table 2: **Sensitivity analysis on temporal resolution of input data.** We show the impact of using daily instead of monthly river flow data, and of using the entire period 2011-2024 at hourly resolution for VRE yield modelling instead of only one meteorological year, on several of the main output parameters of interest in this study. *reg.* = regular; *prud.* = prudent. LOLE = Loss of Load Expectation, measured in share of days per year with capacity deficit.

Supplementary Note 12 Comparative cost analysis of VRE and fossil fuels

We start with a comparative analysis of costs of VRE as compared to fossil fuel-based power generation under regular circumstances (i.e. without focus on the crisis year 2024). We assume that any future deployment of fossil-based power plants in Ecuador would be natural gas-based, based on recent policy decisions that have favoured it over the more expensive fuel oil[24].

Based on the data tabulated in Supplementary Table 3, we calculate the levelised cost of electricity (LCOE) of VRE and compare it to that of natural gas. The levelised cost of electricity (LCOE) is equal to the sum of a power plant's costs over its lifetime, divided by the total electricity output cumulatively generated over its lifetime:

$$\text{LCOE} = \frac{\sum_y \frac{C_y + O_y + F_y}{(1+r)^y}}{\sum_y \frac{E_y}{(1+r)^y}}, \tag{S1}$$

where y represents the year of the plant's lifetime ($0 \leq y \leq Y$, with Y the plant's lifetime); C_y are the capital costs (CAPEX) related to construction of the plant in each year y; O_y are the operational and maintenance costs (OPEX) in each year y; F_y are the fuel costs in each year y (these are zero for renewable resources, like hydro and VRE); E_y is the total electricity generated by the plant in each year y; and r is the discount rate.

Technically, the LCOE is not always a valid metric of comparison, e.g. between baseload and variable resources. However, in our study's results, the VRE is already largely "firmed up" thanks to adapted reservoir hydropower operation. Therefore, in this case, we consider it instructive to compare VRE and fossil fuels on the basis of their LCOE.

We calculate uncertainty ranges of the LCOE based on a range of possible contributions that VRE and additional gas could have in the Ecuadorian power system. For VRE, the main determinant of uncertainty in the LCOE would be the curtailment level; for gas, the main uncertainty would come from the capacity factor (CF), determined by the extent to which hydropower and VRE obviate the need for thermal generation in the power mix. We assume VRE curtailment might realistically range from 0% to (at most) 20% (see **Supplementary Note 8**), whereas we assume that gas capacity factors may range from around 20% to 80% (most thermal power plants in Ecuador have capacity factors in this range, according to historical data[12]).

	C **plant** *\$/kW*	C **grid** *\$/kW*	O **fixed** *\$/kW*	O **variable** *\$/kWh*	F **fuel** *\$/kWh*	CF	Y *years*	r	Curtail-ment	PEF	**LCOE** *\$ct/kWh*
**Solar**	691[26]	92[14]	6.4[26]	-	-	19%	25	12.2%[27]	0% - 20%	1	5.8 - 7.3
**Wind**	1492[26]	109[14]	100[26]	-	-	54%	25	12.2%[27]	0% - 20%	1	6.0 - 7.5
**Gas**	820[28]	-	-	0.004[29]	0.070[30]	20% - 80%	25	8.0%[31]	0%	2.08[32]	8.4 - 11.4

Supplementary Table 3: **Calculation of the LCOE of solar, wind, and gas power plants for Ecuador.** The grid CAPEX (capital expenses, C) for VRE are part of the Model Supply Regions workflow[13] which allowed to create the geospatial dataset in ref.[14] that served as input to our study; the numbers given here represent weighted averages of the necessary grid expansion costs across the investigated locations. The same goes for the VRE capacity factors. The plant CAPEX for wind power are based on the region-specific value for "other South America" from ref.[26]. The fixed OPEX (operational expenses, O) for wind power are based on the most pessimistic side of the ranges given by IRENA in ref.[26]. The fuel cost (F) for gas is based on the unsubsidised cost of 10 USD/MMBTU (million British Thermal Units) cited for Ecuador in ref.[30]; we assume natural gas plants to have a heat rate of 7000 BTU/kWh[32], corresponding to a Primary Energy Factor (PEF) of 2.08. We assume solar and wind power to have higher discount rates than natural gas plants, as the latter are a more established technology in the Ecuadorian context.

Based on these numbers, it appears that VRE would likely allow for cost-competitive electricity production as compared to further thermal-based generation. Even under the most optimistic assumptions for gas and the most pessimistic assumptions for VRE, gas-fired power would not end up at lower levelised costs than solar or wind power.

Aside from the focus on electricity generation costs over a project's lifetime, we can also focus on the specifics of a crisis year like 2024. As our study shows, hydro-backed VRE could substantially reduce the capacity and generation gap in very dry-year settings such as 2024 (comparing scenarios *S1 regular* to *S4 prudent*). In the process, the VRE also reduces overall thermal power generation needs by about one-third. What if we wanted to cover the same capacity gap reductions with natural gas plants? This would imply installing in the order of 600 MW of natural gas plants (as opposed to roughly 1 GW of VRE—see **Supplementary Note 11**), which—if it were to cover, say, the entire generation gap (∼200 GWh)—would be running at a capacity factor of merely 3%-4%, leading to very high costs per unit of electricity from such plants. Increasing the capacity factor to improve their LCOE would not make sense, as this would come down to procuring fossil fuels to displace other fossil fuels from the mix, with no net savings—as opposed to the VRE case, in which displacing thermal power by solar/wind power reduces annual fossil fuel expenses. In other words, an emergency gas power plant only runs in emergencies, whereas VRE plants do not have this constraint. The cost per unit of production of an emergency gas power plant would be so high as to probably render it financially unattractive for investors, unless other mechanisms—e.g. capacity markets—were to be created to remunerate the plant owners for making the capacity available to the system. VRE, on the other hand, would not need such safeguards—while still increasing the system's resilience, as this study shows. Related, another advantage of VRE as compared to thermal power to cover extreme years is that VRE would not suffer from the potential supply chain disruptions and bottlenecks that natural gas and diesel provision may undergo[24,30], which could affect the "emergency procurement" of thermal fuel in crisis times.

Aside from any considerations of overall costs over a project lifetime, this comparison also extends to costs which have to be incurred *during the crisis itself*: to run emergency gas power plants, money needs to be spent *in the moment* to procure the combustible *as soon as possible* (cf. the fact that Ecuador needed to procure a powership barge during the crisis), whereas this is not needed with VRE. This can be illustrated with a simple calculation comparing scenarios *S1 regular* to *S4 prudent*. Under the occurrence of a dry year like 2024 (and based on current electricity demand), the overall demand for thermal power (or imports)—including from Ecuador's existing thermal plants—would have been 3.6 TWh lower in *S4 prudent* than in *S1 regular*, because the integration of VRE displaces demand for fossil fuels and imports. With the fuel cost from Supplementary Table 3, this demand reduction would correspond to avoided fossil fuel expenses of around 260 million USD—a substantial amount, representing roughly 14% of the estimated total economic loss that Ecuador incurred due to the 2024 electricity crisis[33].

Supplementary Note 13 Exploiting wind resources at higher altitudes

As a sensitivity check on the results in our scenarios S1-S4, in which only wind power locations below 2000 m.a.s.l. were considered, we ran an additional scenario S5, equal to S4 but with wind power locations extended to all elevations below 3000 m.a.s.l. As already shown in Supplementary Figure 5 (inset), the higher-altitude locations differ from the lower-altitude ones in two ways. First, the higher-altitude locations reach somewhat higher average capacity factors. This is principally good news for hydro-VRE hybridisation, as less investment in capacity would be needed to reach comparable production. Second, the higher-altitude locations also have a stronger seasonality. This could lead to even more pronounced "extreme-year synergies" with hydropower, which would be good news as it could allow hydropower reservoir levels to recover better during the weak rainy seasons of a crisis year. However, the increased seasonality also means that yield at higher altitudes is somewhat lower during November compared to lower altitudes, which would be bad news as November 2024 was the month with the worst capacity and generation gaps (cf. Figure 6 of the main paper).
Results for S5 clarify how this ambiguity manifests. Supplementary Figure 16 shows, analogously to Figure 4 in the main paper, the seasonal water storage cycle (a) as well as the total duration of shutdowns of Paute-Molino per month and time block (b). These indicators all look substantially improved as compared to S4, with storage levels well above their minimum for most of 2024 and the number of shutdowns reduced to only 8 days (compared to 30 in S4), concentrated in the final two months of the year. These results are expected given the better extreme-year synergy between hydro and wind as compared to S4. Moreover, thanks to the higher average wind yield, only 80% of the VRE capacity needed in S4 would be required for S5, mitigating investment needs for a successful hybridisation.

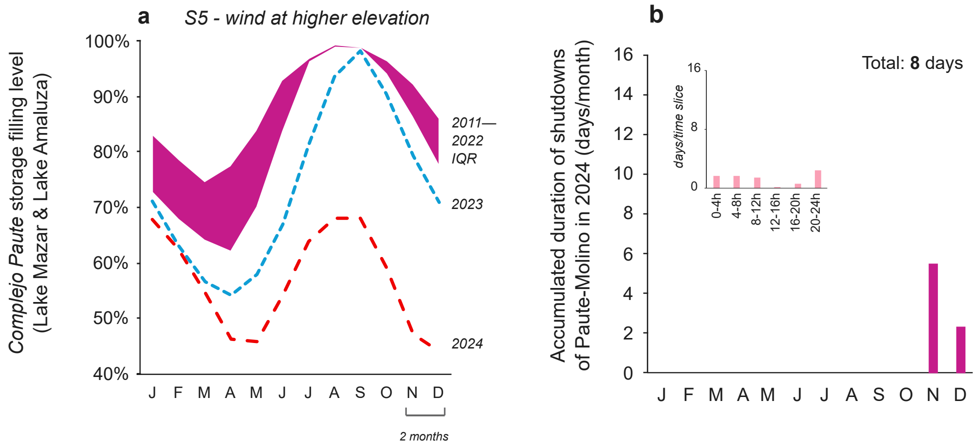

Supplementary Figure 16: **Outcomes for *Complejo Paute* under S5.** Simulation outcomes showing time series of the water storage level in the *Complejo Paute* for scenario S5. Shown are (a) the interquartile range (IQR) of monthly averages for the period 2011-2022, as well as the monthly averages for 2023 (fine blue dashes) and 2024 (rough red dashes), and (b) the accumulated shutdown duration of Paute-Molino in 2024 per month (main graph) and per time block (inset).

However, the outcomes in terms of covering the production shortfall compared to previous years (cf. Figure 5 in the main paper) are, in fact, almost identical to those of S4 (not shown), and so are the capacity and generation gaps in 2024 (cf. Figure 6 in the main paper), shown in Supplementary Figure 17. The reason is that there is a price to pay for the higher wind generation in the critical refill period, which is a lower wind generation in November, at the same time when hydropower generation drops to its yearly minimum and solar is decreasing from its September peak. The result is a slightly increased need for thermal and/or import capacity which nullifies the gains that the further-improved

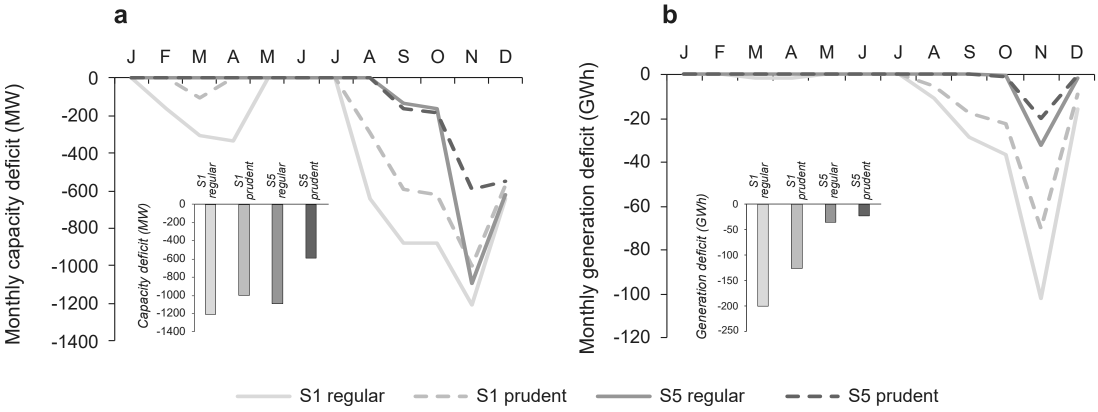

Supplementary Figure 17: **Ecuador's capacity and generation deficit in 2024 under S5.** **a**, The maximum capacity deficit for each month (and the entire year; inset) under S5 compared to S1. **b**, Idem for the corresponding total generation deficit.

storage operation would have brought, as compared to S4.

Thus, whether or not this exploitation of high-altitude wind resources would be worth it in the context of hydro-VRE hybridisation depends on whether the benefits of (i) reducing operational difficulties thanks to avoided plant shutdowns and (ii) avoiding part of the needed VRE capacity buildout, would outweigh the costs of (iii) requiring additional investments per MW to build in high altitudes in the first place, while (iv) potentially needing the same backup capacity to cover capacity gaps in critical periods, as compared to exploiting lower-altitude wind resources. However, the above indicates that it is certainly worth exploring further.

Supplementary References

[1] CELEC. Gráficas de Producción CELEC SUR. URL https://generacioncsr.celec.gob.ec/graficasproduccion/.

[2] Gonzalez-Salazar, M. & Poganietz, W. R. Evaluating the complementarity of solar, wind and hydropower to mitigate the impact of El Niño Southern Oscillation in Latin America. *Renewable Energy* **174**, 453–467 (2021).

[3] Bendix, J., Rollenbeck, R., Göttlicher, D. & Cermak, J. Cloud occurrence and cloud properties in Ecuador. *Climate Research* **30**, 133–147 (2006).

[4] Pineda, L. E., Changoluisa, J. A. & Muñoz, A. G. Early onset of heavy rainfall on the northern coast of Ecuador in the aftermath of El Niño 2015/2016. *Frontiers in Earth Science* **11** (2023).

[5] CELEC. Estudio de Potencial Solar Fotovoltaico del Ecuador (2024). URL https://www.celec.gob.ec/wp-content/uploads/2024/05/Folleto-PROYECTOS-FOTOVOLTAICOS-ECU-2024-2-26-FEB.pdf.

[6] Emck, P. A Climatology of South Ecuador—With special focus on the major Andean ridge as Atlantic-Pacific climate divide (2007). URL https://open.fau.de/items/c9afb786-6208-4fc5-97b4-a058f1d15b63. PhD thesis. Universität Erlangen.

[7] Sterl, S. REVUB: User Manual (2026 version). URL https://github.com/VUB-HYDR/REVUB/blob/master/4_Manual/REVUB_manual.pdf. Vrije Universiteit Brussel.

[8] Sánchez-Juny, M., Larriva, H., Estrella Toral, S. & Sanz-Ramos, M. Modelización de corrientes de turbiedad en embalses. Caso de estudio: Embalse Amaluza (Ecuador). *Ingeniería del Agua* **27**, 253–268 (2023). URL https://polipapers.upv.es/index.php/IA/article/view/20150.

[9] CELEC. Revista 25 años de la presa Daule Peripa (2013). URL https://www.celec.gob.ec/hidronacion/images/PDF/REV_HNA_25_ANOS.pdf.

[10] CELEC. Central Hidroeléctrica Coca Codo Sinclair (n.d.). URL https://www.celec.gob.ec/cocacodo/informacion-tecnica/central-hidroelectrica-coca-codo-sinclair/.

[11] CELEC. Central Hidroeléctrica Delsitanisagua reinició operaciones dos días antes de lo previsto (2019). URL https://www.celec.gob.ec/noticias/central-hidroelectrica-delsitanisagua-reinicio-operaciones-dos-dias-antes-de-lo-previsto/.

[12] CENACE. Informes de Gestión - Informes Anuales (2012-2024). URL https://www.cenace.gob.ec/biblioteca/.

[13] Sterl, S. *et al.* An all-Africa dataset of energy model "supply regions" for solar photovoltaic and wind power. *Scientific Data* **9**, 664 (2022).

[14] Sterl, S. Solar PV and wind power Model Supply Region (MSR) dataset as energy model input for countries in Central and South America (2024). URL https://doi.org/10.5281/zenodo.10650822.

[15] Arderne, C., Zorn, C., Nicolas, C. & Koks, E. Predictive mapping of the global power system using open data. *Scientific Data* **7**, 10 (2020).

[16] Arderne, C. GridFinder - Global Energy Infrastructure. URL https://gridfinder.rdrn.me/.

[17] GADM. Global Administrative Areas v4.1 (2026). URL https://gadm.org/data.html.

[18] CELEC. Informe de Rendición de Cuentas 2018 - Unidad de Negocio GENSUR (2019). URL https://www.celec.gob.ec/gensur/rendicion-cuentas/rendicion-de-cuentas-2018/.

[19] Sterl, S., Liersch, S., Koch, H., van Lipzig, N. P. & Thiery, W. A new approach for assessing synergies of solar and wind power: Implications for West Africa. *Environmental Research Letters* **13**, 094009 (2018).

[20] Nyenah, E., Sterl, S. & Thiery, W. Pieces of a puzzle: solar-wind power synergies on seasonal and diurnal timescales tend to be excellent worldwide. *Environmental Research Communications* **4**, 055011 (2022).

[21] Sterl, S. REVUB model input for the paper "Variable renewables fortify Ecuador's power system against recurrences of drought-driven energy crises" (2026). URL `https://doi.org/10.5281/zenodo.15854447`.

[22] Caceres, A. L., Jaramillo, P., Matthews, H. S., Samaras, C. & Nijssen, B. Hydropower under climate uncertainty: Characterizing the usable capacity of Brazilian, Colombian and Peruvian power plants under climate scenarios. *Energy for Sustainable Development* **61**, 217–229 (2021).

[23] IRENA. Planning for the renewable future: Long-term modelling and tools to expand variable renewable power in emerging economies (2017). URL `https://www.irena.org/-/media/Files/IRENA/Agency/Publication/2017/IRENA_Planning_for_the_Renewable_Future_2017.pdf`. International Renewable Energy Agency, Abu Dhabi.

[24] Orozco, M. Gobierno ordena importar gas natural y subir producción de Campo Amistad para afrontar estiaje de septiembre de 2025 (2025). URL `https://www.primicias.ec/economia/compra-gas-natural-produccion-campo-amistad-estiaje-cortes-luz-95510/`. Primacias.

[25] prensa.ec. Crisis energética en Ecuador: ¿Soluciones a corto plazo o improvisación tardía? (2024). URL `https://prensa.ec/noticias/crisis-energetica-en-ecuador-soluciones-a-corto-plazo-o-improvisacion-tardia/`.

[26] IRENA. Renewable Power Generation Costs 2024 (2025). URL `https://www.irena.org/Publications/2025/Jun/Renewable-Power-Generation-Costs-in-2024`. International Renewable Energy Agency, Abu Dhabi.

[27] IRENA. The cost of financing for renewable power (2023). URL `https://www.irena.org/Publications/2023/May/The-cost-of-financing-for-renewable-power`. International Renewable Energy Agency, Abu Dhabi.

[28] EIA. U.S. construction costs rose slightly for solar and wind, dropped for natural gas in 2022 (2024). URL `https://www.eia.gov/todayinenergy/detail.php?id=63485`. U.S. Energy Information Administration.

[29] EIA. Average Power Plant Operating Expenses for Major U.S. Investor-Owned Electric Utilities, 2013 through 2023 (Mills per Kilowatthour) (2024). URL `https://www.eia.gov/electricity/annual/html/epa_08_04.html`. U.S. Energy Information Administration.

[30] Orozco, M. Ecuador comprará gas natural para subir la oferta de electricidad (2023). URL `https://www.primicias.ec/noticias/economia/celec-comprara-gas-natural-para-subir-la-oferta-de-electricidad/`. Primacias.

[31] Carvajal, P. E. *et al.* Large hydropower, decarbonisation and climate change uncertainty: Modelling power sector pathways for Ecuador. *Energy Strategy Reviews* **23**, 86–99 (2019).

[32] EIA. Use of natural gas-fired generation differs in the United States by technology and region) (2024). URL `https://www.eia.gov/todayinenergy/detail.php?id=61444`. U.S. Energy Information Administration.

[33] Astudillo, E. Ecuador, un año después de la crisis eléctrica: ¿se pueden evitar nuevos apagones? (2025). URL `https://www.bloomberglinea.com/latinoamerica/ecuador/ecuador-un-ano-despues-de-la-crisis-electrica-se-pueden-evitar-nuevos-apagones/`. Bloomberg Línea.
