# Supplementary material for: Variable renewables fortify Ecuador’s power system against recurrences of drought-driven energy crises
Source: Nat Water. 2026 Apr 7;4(5):571–85. doi: 10.1038/s44221-026-00617-w (PMC13197224; doi:10.1038/s44221-026-00617-w)
Supplement: Supplementary file 2 — Reporting Summary [file 44221_2026_617_MOESM2_ESM.pdf]

nature portfolio

Corresponding author(s): Sebastian Sterl

Last updated by author(s): Feb 26, 2026

Reporting Summary

Nature Portfolio wishes to improve the reproducibility of the work that we publish. This form provides structure for consistency and transparency in reporting. For further information on Nature Portfolio policies, see our Editorial Policies and the Editorial Policy Checklist.

Statistics

For all statistical analyses, confirm that the following items are present in the figure legend, table legend, main text, or Methods section.

n/a	Confirmed	
☐	☒	The exact sample size (*n*) for each experimental group/condition, given as a discrete number and unit of measurement
☒	☐	A statement on whether measurements were taken from distinct samples or whether the same sample was measured repeatedly
☒	☐	The statistical test(s) used AND whether they are one- or two-sided<br>*Only common tests should be described solely by name; describe more complex techniques in the Methods section.*
☒	☐	A description of all covariates tested
☐	☒	A description of any assumptions or corrections, such as tests of normality and adjustment for multiple comparisons
☐	☒	A full description of the statistical parameters including central tendency (e.g. means) or other basic estimates (e.g. regression coefficient) AND variation (e.g. standard deviation) or associated estimates of uncertainty (e.g. confidence intervals)
☒	☐	For null hypothesis testing, the test statistic (e.g. *F*, *t*, *r*) with confidence intervals, effect sizes, degrees of freedom and *P* value noted<br>*Give P values as exact values whenever suitable.*
☒	☐	For Bayesian analysis, information on the choice of priors and Markov chain Monte Carlo settings
☒	☐	For hierarchical and complex designs, identification of the appropriate level for tests and full reporting of outcomes
☒	☐	Estimates of effect sizes (e.g. Cohen's *d*, Pearson's *r*), indicating how they were calculated

Our web collection on statistics for biologists contains articles on many of the points above.

Software and code

Policy information about availability of computer code

Data collection	The study made use of the REVUB model (version 1.1). The model is Python-based, open-source, and free, and can be accessed via https://github.com/VUB-HYDR/REVUB. It has been used in various other peer-reviewed studies in the past, and is fully documented in the corresponding Manual available on the GitHub page (https://github.com/VUB-HYDR/REVUB/blob/master/4_Manual/REVUB_manual.pdf).
Data analysis	Data was analysed using standard Python and spreadsheet-based calculation tools.

For manuscripts utilizing custom algorithms or software that are central to the research but not yet described in published literature, software must be made available to editors and reviewers. We strongly encourage code deposition in a community repository (e.g. GitHub). See the Nature Portfolio guidelines for submitting code & software for further information.

Data

Policy information about availability of data

All manuscripts must include a data availability statement. This statement should provide the following information, where applicable:

- Accession codes, unique identifiers, or web links for publicly available datasets
- A description of any restrictions on data availability
- For clinical datasets or third party data, please ensure that the statement adheres to our policy

We are grateful to IRENA for making available the Model Supply Regions workflow (https://github.com/SPLATteam/Model-Supply-Regions-MSR-Toolset) which allowed to create the Model Supply Regions dataset for Central and South America (https://doi.org/10.5281/zenodo.10650822); to CELEC for making available high-

resolution hydrological and energy-related data for its CELEC SUR business unit (https://generacioncsr.celec.gob.ec/graficasproduccion/); and to CENACE for the hydrological and energy-related data available in its annual reports covering nearly the entire period investigated in this paper (https://www.cenace.gob.ec/biblioteca/). The REVUB model input files used to run this study's scenarios are made available on Zenodo (https://doi.org/10.5281/zenodo.15854448). All supplementary data sources not cited in this paper are provided therein.

Research involving human participants, their data, or biological material

Policy information about studies with human participants or human data. See also policy information about sex, gender (identity/presentation), and sexual orientation and race, ethnicity and racism.

Reporting on sex and gender	n/a
Reporting on race, ethnicity, or other socially relevant groupings	n/a
Population characteristics	n/a
Recruitment	n/a
Ethics oversight	n/a

Note that full information on the approval of the study protocol must also be provided in the manuscript.

Field-specific reporting

Please select the one below that is the best fit for your research. If you are not sure, read the appropriate sections before making your selection.

☐ Life sciences ☐ Behavioural & social sciences ☒ Ecological, evolutionary & environmental sciences

For a reference copy of the document with all sections, see nature.com/documents/nr-reporting-summary-flat.pdf

Ecological, evolutionary & environmental sciences study design

All studies must disclose on these points even when the disclosure is negative.

Study description	We study here if, and how, synergetic hydro-VRE operation could have helped Ecuador weather its 2024 energy crisis, and the role of reservoir management therein. In other words: If Ecuador opted for large-scale VRE buildout, supported by hydropower, could it avoid reoccurrences of drought-induced energy crises in the future?
Research sample	The research covers Ecuador's existing power generation fleet (hydropower, thermal power and wind power) and investigates scenarios for its drought resilience through addition of solar and wind power and adaptation of the operation of the existing hydropower fleet.
Sampling strategy	Not applicable since this is a modelling study of Ecuador's power system
Data collection	Data was collected from various openly available datasets, as described in the Data Availability statement.
Timing and spatial scale	The time period covered in the study concerns 2011-2024. The spatial scale of the investigation is Ecuador, although the study has implications beyond Ecuador's borders.
Data exclusions	The period before 2011 was not taken into account, since Ecuador's largest hydropower storage lake was not yet in operation then. Hydropower storage plays a crucial role in the study.
Reproducibility	The modelling study can be replicated using the REVUB model and the input dataset provided on Zenodo.
Randomization	Not applicable since this is a modelling study of Ecuador's power system
Blinding	Not applicable since this is a modelling study of Ecuador's power system

Did the study involve field work? ☐ Yes ☒ No

Reporting for specific materials, systems and methods

We require information from authors about some types of materials, experimental systems and methods used in many studies. Here, indicate whether each material, system or method listed is relevant to your study. If you are not sure if a list item applies to your research, read the appropriate section before selecting a response.

Materials & experimental systems

n/a	Involved in the study
☒	☐ Antibodies
☒	☐ Eukaryotic cell lines
☒	☐ Palaeontology and archaeology
☒	☐ Animals and other organisms
☒	☐ Clinical data
☒	☐ Dual use research of concern
☒	☐ Plants

Methods

n/a	Involved in the study
☒	☐ ChIP-seq
☒	☐ Flow cytometry
☒	☐ MRI-based neuroimaging

Plants

Seed stocks	n/a
Novel plant genotypes	n/a
Authentication	n/a
